# The diversity and evolution of cell cycle regulation in alpha-proteobacteria: a comparative genomic analysis

**DOI:** 10.1186/1752-0509-4-52

**Published:** 2010-04-28

**Authors:** Matteo Brilli, Marco Fondi, Renato Fani, Alessio Mengoni, Lorenzo Ferri, Marco Bazzicalupo, Emanuele G Biondi

**Affiliations:** 1 Department of Evolutionary Biology, University of Florence, via Romana, 17, Florence, Italy; 2 Laboratoire de Biométrie et Biologie Evolutive, UMR CNRS 5558, Université Lyon 1, 43, bvd du 11 novembre, Lyon, France

## Abstract

**Background:**

In the bacterium *Caulobacter crescentus*, CtrA coordinates DNA replication, cell division, and polar morphogenesis and is considered the cell cycle master regulator. CtrA activity varies during cell cycle progression and is modulated by phosphorylation, proteolysis and transcriptional control. In a phosphorylated state, CtrA binds specific DNA sequences, regulates the expression of genes involved in cell cycle progression and silences the origin of replication. Although the circuitry regulating CtrA is known in molecular detail in *Caulobacter*, its conservation and functionality in the other alpha-proteobacteria are still poorly understood.

**Results:**

Orthologs of *Caulobacter *factors involved in the regulation of CtrA were systematically scanned in genomes of alpha-proteobacteria. In particular, orthologous genes of the *divL-cckA-chpT-ctrA *phosphorelay, the *divJ*-*pleC*-*divK *two-component system, the *cpdR*-*rcdA*-*clpPX *proteolysis system, the methyltransferase *ccrM *and transcriptional regulators *dnaA *and *gcrA *were identified in representative genomes of alpha-proteobacteria. CtrA, DnaA and GcrA binding sites and CcrM putative methylation sites were predicted in promoter regions of all these factors and functions controlled by CtrA in all alphas were predicted.

**Conclusions:**

The regulatory cell cycle architecture was identified in all representative alpha-proteobacteria, revealing a high diversification of circuits but also a conservation of logical features. An evolutionary model was proposed where ancient alphas already possessed all modules found in *Caulobacter *arranged in a variety of connections. Two schemes appeared to evolve: a complex circuit in *Caulobacterales *and *Rhizobiales *and a simpler one found in *Rhodobacterales*.

## Background

Living cells continuously receive and process signals coming from their environment, and by integrating this information into their own internal state, are able to react with appropriate responses which coordinate each function in the cell in order to divide and produce progeny. Regulation of cell cycle progression needs to be a robust but versatile process that integrates different exogenous and endogenous signals and that guarantees fidelity and controlled progression throughout the different phases.

Bacteria have evolved different regulation systems for cell cycle coordination, probably due to different ecological and evolutionary constraints [1,2]. Alpha-proteobacteria subdivision is a very heterogeneous group of bacteria and includes symbionts of plants (Rhizobia), pathogens for animals (*Brucella*, *Rickettsia*) and plants (*Agrobacterium*), photosynthetic bacteria (*Rhodobacter*) and also several genera metabolizing C1-compounds (*Methylobacterium*).

Together with this diversity of life styles and ecological niches, the alpha-proteobacteria subdivision is also one of the bacterial groups in which cell cycle regulation has been studied in more detail and one of its members, *Caulobacter crescentus*, has recently become a model organism in these studies [3-6]. In this organism each cell division is asymmetric—producing two functionally and morphologically different cells, the replicating "stalked" cell type and the vegetative "swarmer" type. After each initiation of DNA replication, the replication fork is kept blocked so that the *Caulobacter *cell cycle can follow a pattern of once-and-only-once replication per division (G1, S, and G2 phases are temporally distinguished).

Many factors are known to regulate cell cycle progression and most of them are members of the family of two-component signal transduction proteins, comprised of histidine kinases and their response regulator substrates [6]. Among those proteins CtrA is the master regulator of the *Caulobacter *cell cycle, an essential response regulator whose activity as a transcription factor varies as a function of the cell cycle [7-9]. CtrA controls various functions during cell cycle progression by activating or repressing gene expression. CtrA also blocks the initiation of DNA replication through binding of the replication origin [7]. Among genes regulated by CtrA we can find those involved in cell division (*ftsZ*, *ftsA*, *ftsQ *and *ftsW*), the protease encoding gene *clpP *which is essential in *Caulobacter*, the DNA methylase gene *ccrM*, flagellar biogenesis genes, stalk biogenesis regulatory genes, pili biogenesis genes such as *pilA*, and chemotaxis genes [10-15].

CtrA activity and stability varies during the cell cycle. Oscillation of CtrA levels, peaking at the predivisional stage before cell division, is achieved by different mechanisms: transcription, proteolysis and phosphorylation control as discussed in detail below.

DnaA and GcrA, and the DNA methyltransferase CcrM are involved in controlling *ctrA *transcription [11,16]. DnaA is a key element in cell cycle regulation because it is required for the initiation of DNA replication; it also controls the transcription of about 40 genes involved in nucleotide biogenesis, cell division, and polar morphogenesis [17,18], and it activates the transcription of the *gcrA *gene [19]. GcrA controls the transcription of *ctrA *and genes involved in DNA metabolism and chromosome segregation, including those encoding DNA gyrase, DNA helicase, DNA primase, and DNA polymerase III [19]. As a consequence of this genetic circuit, CtrA accumulates out-of-phase with GcrA [19]. The transcriptional loop of *ctrA *is closed by CcrM. CtrA activates the transcription of *ccrM*, which encodes for a DNA methyltransferase whose abundance is cell cycle dependent. CcrM is able to activate *dnaA *promoter region through methylation, closing the positive feedback composed by CtrA, DnaA and GcrA.

A second essential regulatory control on CtrA is carried out by phosphorylation. In fact, CtrA must be phosphorylated to bind DNA and its phosphorylation depends on cell cycle progression. An essential phosphorelay, composed of the hybrid histidine kinase CckA and the histidine phosphotransferase ChpT, is responsible for CtrA phosphorylation [20,21].

DivK, which is a response regulator, plays an essential role as a positive regulator of cell cycle progression because when phosphorylated, it indirectly inactivates CtrA and thus promotes DNA replication. Two histidine kinases are known to interact with DivK: PleC and DivJ [22-25]. Bacterial histidine kinases can have alternatively both kinase and phosphatase activities and these opposite activities are modulated by conformational changes of the protein [26]. A null *Caulobacter pleC *mutant produces almost symmetric cells at the division and displays abnormal polar development. The DivJ histidine kinase plays a role in controlling the length and location of the stalk and cell division. PleC and DivJ are considered the principal phosphatase and kinase, respectively, of DivK and they are in opposite locations during cell cycle progression [27,28]. DivJ activity is also positively controlled by the TacA/SpmX pathway, which is transcriptionally activated by CtrA [10,29].

ChpT also transfers the phosphate to a second response regulator named CpdR, which, together with RcdA, is a factor involved in CtrA proteolysis mediated by ClpP-ClpX protease [30-32]. CtrA is degraded at the stalked pole at the G1/S transition when the origin of replication needs to be cleared and also in the stalked compartment, where initiation of DNA replication occurs immediately after cell division [33,34].

**Figure 1:**
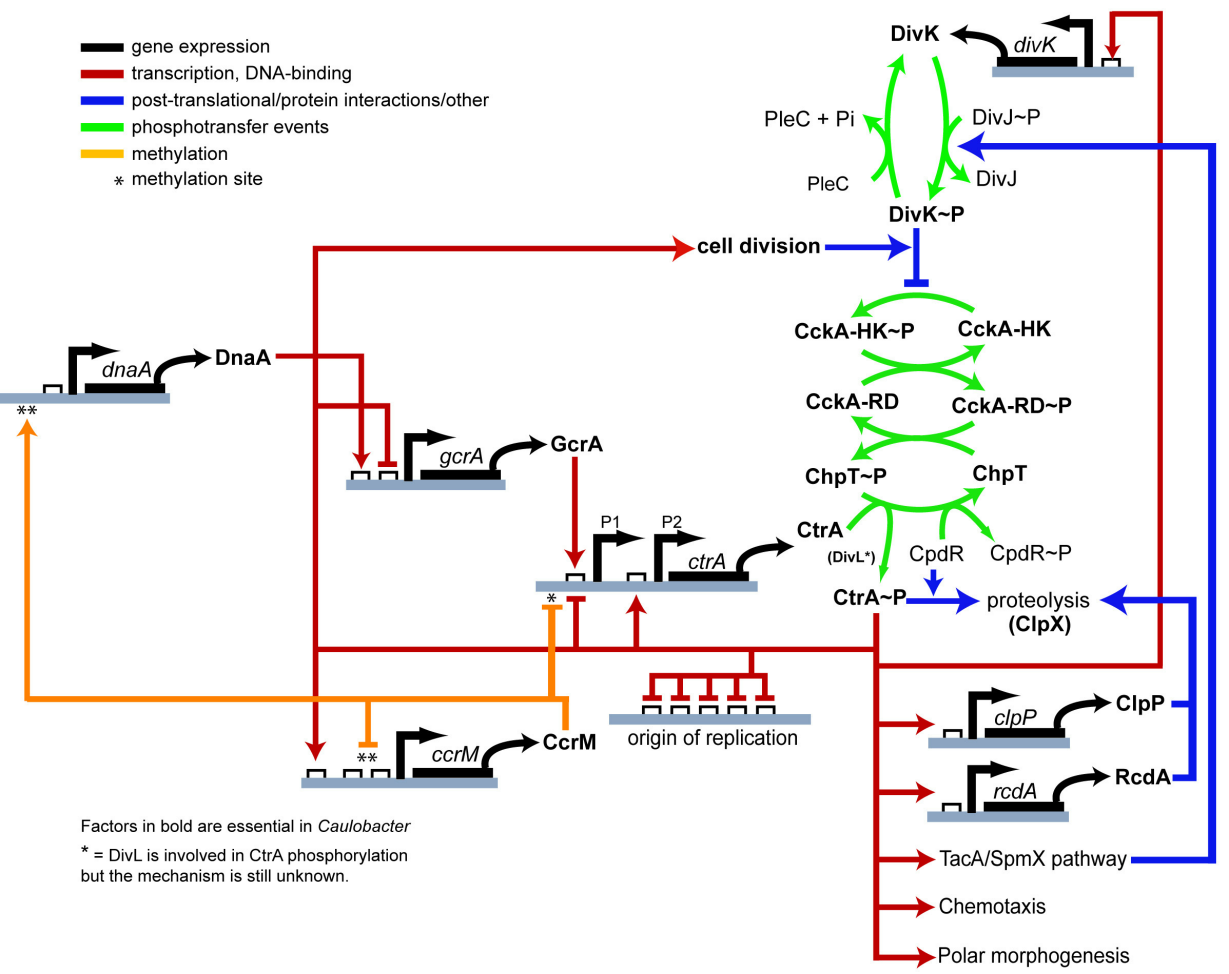
**Cell cycle regulation in *Caulobacter***. Scheme of cell cycle regulation in *Caulobacter *with all factors analyzed in this paper. See the "introduction" for details.

All these regulations are schematized in Figure 1 where we illustrate the multilevel regulation of the *Caulobacter *cell cycle. Two main oscillators are working during cell cycle progression: (i) the transcriptional and epigenetic circuit (CtrA-DnaA-GcrA-CcrM); (ii) the phosphorylation/proteolysis and transcription circuit (CckA-CtrA-DivK). The latter also involves coordination of CtrA proteolysis and cell division through regulation of DivK activity.

Several of these regulatory mechanisms are at least partially redundant, and it has been demonstrated that only phosphorylation of CtrA is indispensable during cell cycle progression; in fact, cell cycle regulated transcription of *ctrA *can be substituted by constitutive transcription [20] and proteolysis can also be removed.

It has recently been demonstrated that asymmetric division also takes place in *Agrobacterium tumefaciens*, *Sinorhizobium meliloti *and *Brucella abortus *[35], indicating that at least some of the features governing cell cycle progression in *Caulobacter *might also be present in other species. For example, in *Rhodobacter capsulatus*, CtrA and CckA are not essential but are required for the expression of the GTA, a system for genetic exchanges [36,37]. CtrA in *Brucella *controls cellular events similar to those controlled by CtrA in *Caulobacter*, but through a direct effect on different targets [38]. Moreover CtrA from *Caulobacter *is able to bind the *B. abortus ccrM *promoter in vitro [39] and CtrA from *B. abortus *has been shown to bind promoters of *ccrM, pleC*, *rpoD, ftsE *and *minC *but not *divK, ftsZ *or the origin of replication, that are known CtrA targets in *Caulobacter *[38]. In *Silicibacter pomeroy *three known mutants affect the motility, two of which are *cckA*, *ctrA *[40].

*ccrM *is essential and cell cycle regulated in *A. tumefaciens *[41], as observed in *B. abortus*, where *ccrM *is also essential and its overexpression impairs proper intracellular replication in murine macrophages [39], revealing a link between cell cycle and pathogenetic activity.

We hereby undertook a comparative and integrative analysis of factors controlling cell cycle regulation, and the regulatory connections existing between them, looking for orthologous genes of factors that are involved in controlling CtrA, the master regulator, in *C. crescentus*. We also analyzed CtrA-regulated functions in 37 representative genomes of alphas. At the same time, binding sites of DnaA, GcrA and the presence of putative methylation sites of CcrM were also suggested through bioinformatic analysis. All this information was used to reconstruct the architecture of CtrA regulation throughout the phylogenetic tree of alpha-proteobacteria to reveal evolutionary trends and insights into this complex regulation.

## Results

### Evolutionary scheme of alpha-proteobacteria

**Figure 2:**
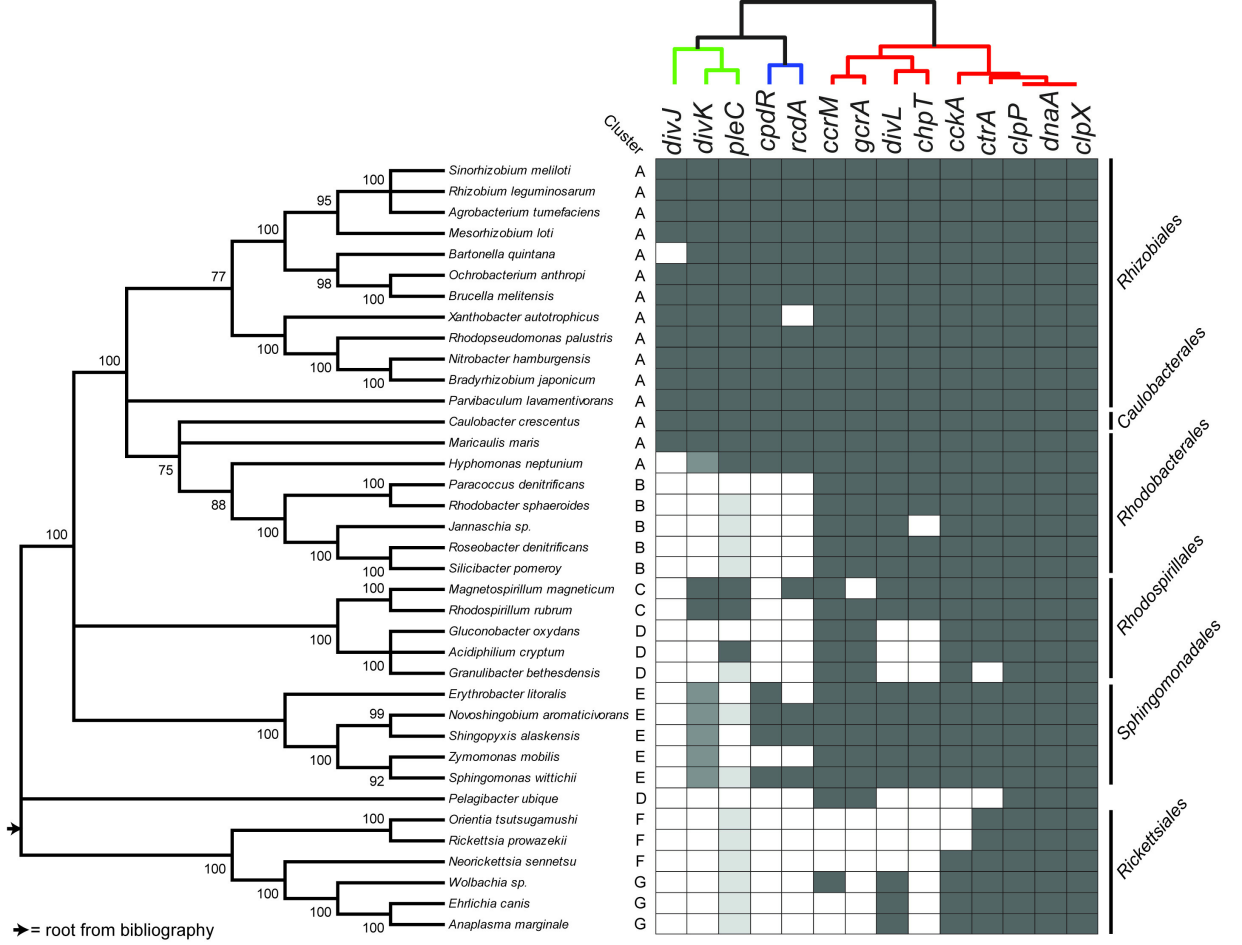
**Orthologous genes in alpha-proteobacteria**. Presence (black) or absence (white) of genes (found using bidirectional best blast hit, BBH) described in Figure 1 and involved in cell cycle regulation in the alpha-proteobacterial genomes. Proteins are indicated in the uppermost row and they are ordered on the basis of their co-occurrence patterns (see Material and Methods). We show here a reduced dataset comprising one species for each considered genus, for a total of 37 out of 65 alpha-proteobacteria analyzed (the complete results are listed in Table S2, Additional file 4). Organisms are ordered on the basis of their phylogenetic relationships as assessed by using a 5000-residue long concatamer of universal proteins [65], the Neighbor-Joining method with a Dayhoff evolutionary model and 100 bootstrap replicates. A tree representation where nodes with a statistical support of less than 75% have been collapsed (Figure 2, left) was chosen; Dark gray cells indicate the presence of a BBH with respect to the protein corresponding to that column. Medium dark gray cells correspond to DivK proteins in those organisms that have an ambiguous position in the phylogenetic tree (Additional file 3, Figure S1); light gray cells correspond to organisms that have PleC but where DivK is absent or in doubt.

To construct a robust phylogeny of alpha-proteobacteria, a dataset of eight universal proteins from the Ribosomal Database Project was downloaded. Proteins were aligned separately and then the alignments were concatenated, resulting in an alignment of 5056 amino acids that has been used to construct a Neighbor-Joining tree (see Methods) (Figure 2). A comparison with previous phylogenetic trees of alpha-proteobacteria [42] suggested that the tree root lies in the branch connecting the *Rickettsiales *to the other alpha-proteobacteria. Unlike the work of Gupta and Mok (2007), our tree shows *Sphingomonadales *branching off after the *Rickettsiales *and followed by the branching of *Rhodospirillales*, whereas in other works the latter branched first.

### Clusterization of alpha-proteobacteria based on the orthology of cell cycle genes

By using the bidirectional best hit (BBH) approach (see Material and Methods section) on 65 available genomes of alpha-proteobacteria (Additional file 1, Table S1; legends of additional Figures and Tables are in Additional file 2) we obtained a list of genes that are orthologous to the 14 genes involved in the *Caulobacter *cell cycle, and the results are reported in Figure 2 (see also Additional file 3, Figure S1 and Additional file 4, Table S2).

ClpX, ClpP and DnaA are present in all alphas studied, but surprisingly all other proteins analyzed can be absent in several alphas. Transcription factors, GcrA and CtrA, the DNA methyl-transferase CcrM and the hybrid histidine kinase CckA are present in most of the alpha-proteobacteria. Other modules, such as those of the DivJ-PleC-DivK two-component system, are present only in clusters A and C of alpha-proteobacteria.

Genes with similar phylogenetic profiles (genes co-occurring in different genomes) are often functionally related [43] justifying the use of our profiles to investigate possible functional relationships; the dendrogram obtained describes how similar the profiles of different genes are (see Methods and upper part of Figure 2) and it confirms the functional association between *divJ*, *pleC *and *divK *(encoding the two component system negatively regulating CckA activity), and between *cpdR *and *rcdA*, whose products are involved in CtrA proteolysis. Weaker and possibly new functional associations concern the gene pair *ccrM*/*gcrA *and *divL*/*chpT*. In addition, we visually inspected the phylogenetic profiles of these genes between organisms, identifying seven groups (from A to G, see Figure 2). This classification, based on orthology, will be used as a reference in the following sections.

Cluster A includes *Rhizobiales*, *Caulobacterales *and several *Rhodobacterales*, and is composed of the largest number of sequenced genomes; this cluster is characterized by a nearly identical conservation of factors found in *Caulobacter*. Although similarities are evident in this cluster, a deeper analysis revealed substantial differences that will be discussed in the next sections.

Cluster B, including other *Rhodobacterales*, shows a substantial difference compared to cluster A; in fact, both DivJ-PleC-DivK and RcdA-CpdR systems are missing.

*Magnetospirillum *and *Rhodospirillum*, which are closely related, are the two members of cluster C. This cluster is characterized by the presence of the PleC-DivK system since DivJ is missing and by an almost complete loss of the CpdR-RcdA system although *Magnetospirillum *possesses an *rcdA *orthologous gene.

Clusters D (*Rhodospirillales*) and F (part of *Rickettsiales*), even though they are separated in the tree reported in Figure 2, share common features: i. members of the CckA-ChpT-CtrA phosphorelay are missing at different degrees (*Granulibacter *and *Pelagibacter *do not show even *ctrA *orthologs); ii. DivJ-PleC-DivK and RcdA-CpdR systems are missing. Despite these similarities, the two groups diverge for the presence of CcrM and GcrA in group D.

Organisms belonging to cluster E (*Sphingomonadales*) show conservation of the phosphorelay CckA-ChpT-CtrA and also often possess factors required for the temporally and spatially regulated proteolysis of CtrA, such as CpdR and RcdA. However cluster E is characterized by degeneration or a complete loss of the DivJ-PleC-DivK regulation system. DivK or PleC orthologs can be found in several organisms of this subgroup although their phylogeny often significantly deviate from the phylogenetic tree of housekeeping genes (Additional file 5, Figure S2).

Finally, group G (remaining *Rickettsiales*), composed mainly of pathogens, has few of the factors involved in *Caulobacter *cell cycle progression regulation. It is however interesting to find a CckA-CtrA system whereas ChpT orthologs cannot be found using the BBH approach.

### CtrA regulon in alpha-proteobacteria

The regulatory circuit that controls cell cycle progression in *Caulobacter *is also composed of crucial transcriptional connections, such as CtrA control on *divK *and the CtrA-DnaA-GcrA-CcrM circuit. This transcriptional regulation level is discussed in this section and the following. In particular, results obtained for the prediction of the CtrA regulon in alpha-proteobacteria are described here.

Laub and collaborators [13,44] were able to identify a set of genes plausibly constituting the CtrA regulon in *Caulobacter *by combining varying evidence: 116 genes were identified through chromatin immunoprecipitation using phosphorylated CtrA; 88 genes were identified as CtrA-dependent for normal expression levels, and 69 as cell cycle regulated in a transcriptome analysis encompassing one complete cell cycle round. The 54 genes within the overlap of all three data sets were identified as members of the CtrA cell cycle regulon, and were used here to build the position weight matrix (PWM) of CtrA binding sites. Upstream sequences of these 54 genes were retrieved and used to find enriched sequence motifs using AlignAce [45]. The PWM obtained (Additional file 6, Table S3) corresponds to a 16-mer containing the known CtrA binding motif and was used in a sliding window approach on a non-redundant subset of the genomes used in this work. An output was obtained where genes in a given genome have a score based on the presence of CtrA sequence motifs in the region comprised of 100 nucleotides within the coding sequence to 400 nucleotides upstream of the start codon (see Methods for details).

Is the CtrA PWM, based on *Caulobacter *data, valid for all the alphas analyzed here? One might speculate that if a *ctrA *gene taken from an alpha is able to complement deletion in *Caulobacter*, the PWM built on *Caulobacter ctrA*-controlled genes would also be valid for the bacterium where the complementing *ctrA *comes from. It has been shown that the *ctrA *gene from *R. prowazekii*, named *czcR*, is able to complement the deletion of *ctrA *in *Caulobacter*, confirming that the functionality (that is, the binding site) is conserved between *Rickettsia *and *Caulobacter *[46]. Moreover other *ctrA *genes from species taxonomically closer to *Caulobacter*, such as *S. meliloti*, are able to complement the *ctrA *deletion in *Caulobacter *(Biondi, unpublished data). Considering the phylogeny of alphas and positions in the tree of *R. prowazekii *and *S. meliloti*, it is reasonable to consider that the CtrA binding site might be substantially conserved across the alphas.

**Figure 3:**
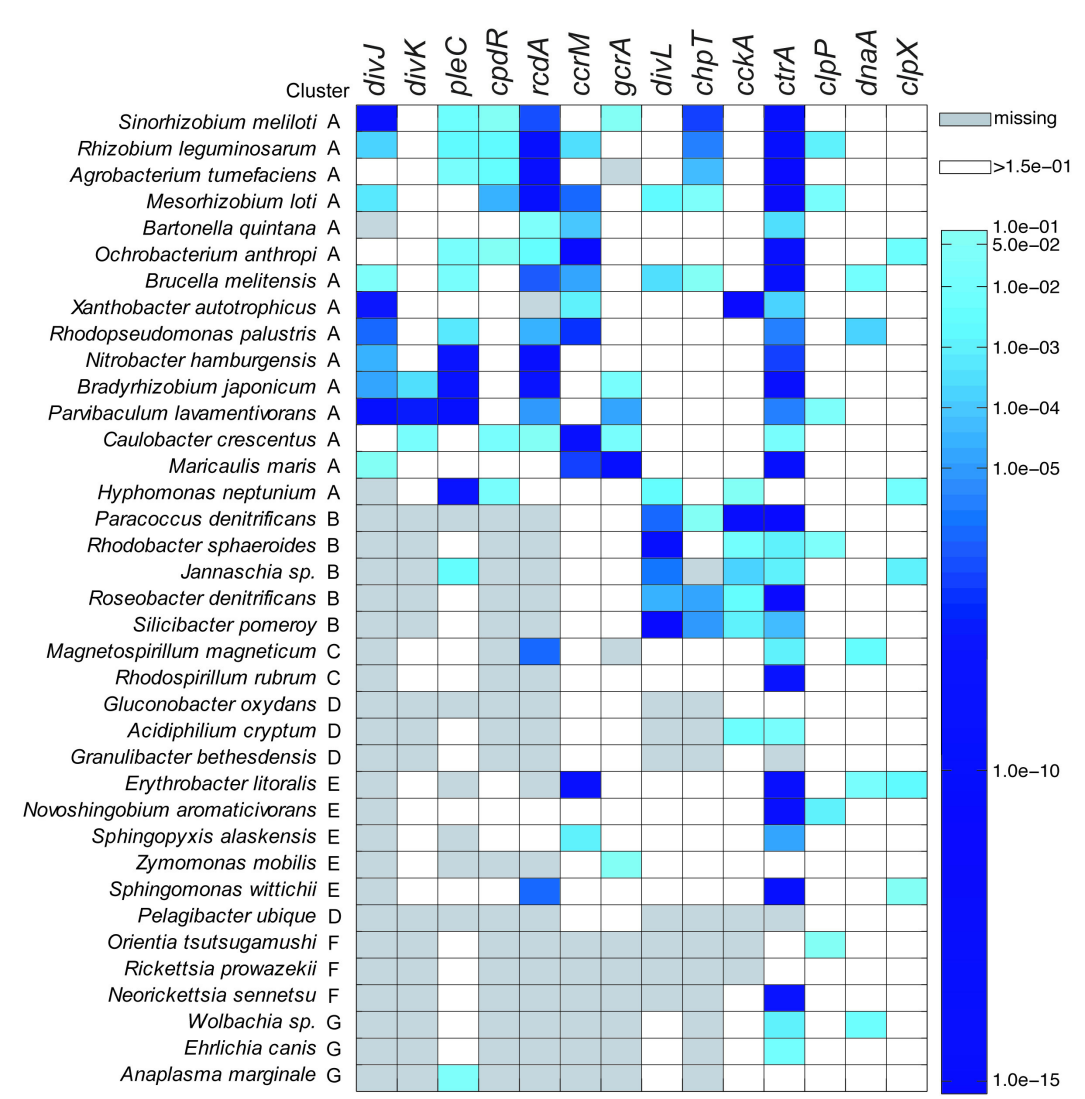
**Transcriptional control of CtrA on cell cycle genes**. Transcriptional control of CtrA on factors involved in regulation of cell cycle progression (see also Additional file 7, Table S4). Color bar represents p-values of the Z-score transformation of motif scores (Methods section for details).

**Figure 4:**
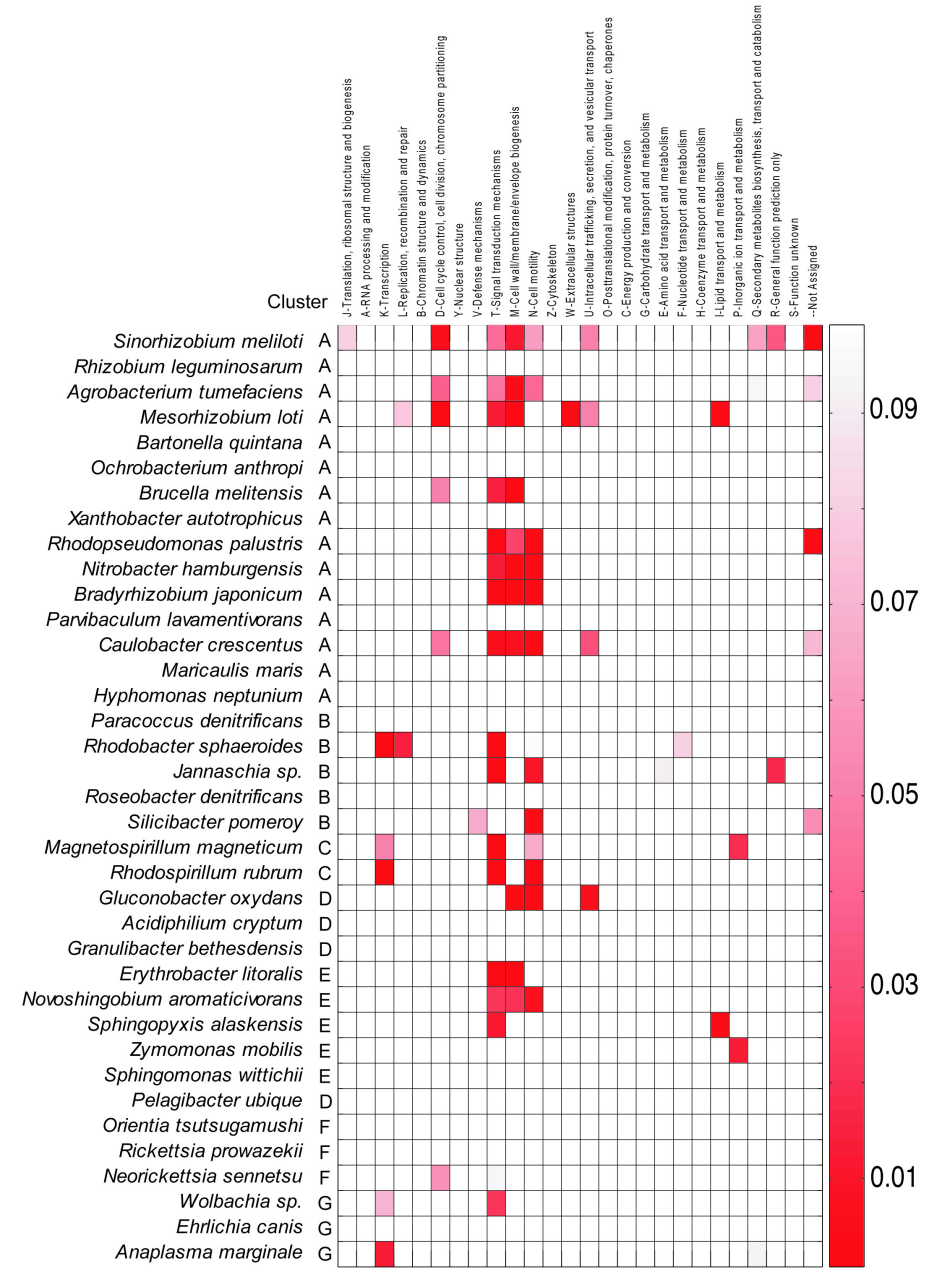
**Functions controlled by CtrA among alpha-proteobacteria**. Enrichment of genes controlled by CtrA in COG categories (see also Additional file 8, Table S5). The scale corresponds to the p-value of the functional enrichment calculated as described in the Material and Methods. The p-value is inversely correlated to the strength of the functional enrichment of each regulon, i.e. a lower p-value indicates stronger enrichment.

Two kinds of results from this analysis are shown here: (i) CtrA target genes belonging to our starting dataset of cell cycle related genes (Figure 3) and (ii) enrichment of COG (clusters of orthologous groups of proteins) categories of genome-wide CtrA regulons for each genome analyzed (Figure 4).

In Figure 3 (see also Additional file 7, Table S4) we show the p-values for the presence of CtrA binding sites upstream of analyzed genes. CtrA controls the transcription of several genes involved in regulation of cell cycle progression, including itself in most of the alphas analyzed (86%). Moreover, the number of genes controlled by CtrA is maximal in cluster A. In this cluster CtrA controls at least one gene of each of the following systems: DivJ-DivK-PleC, CpdR-RcdA-ClpPX and GcrA-DnaA-CcrM. Several genes showed evolutionary conservation of CtrA control among members of cluster A, such as DivJ, RcdA and CcrM. The second important result arising from the analysis shown in Figure 3 was that in Cluster B, where the DivJ-PleC-DivK system is missing, CtrA controls both CckA and DivL.

Each genome-wide regulon of CtrA was defined as the list of genes with a Z-score (see Methods) ≥2 (corresponding to a p-value of ca. 0.023) in an organism. In Figure 4 (see also Additional file 8, Table S5), predicted regulons of CtrA were analyzed for functional enrichment (percentage of genes in a COG category controlled by CtrA) in genes belonging to functional categories. Most enriched categories were *Signal transduction mechanisms *(enriched in 15 organisms), *Cell wall/membrane/envelope biogenesis *(enriched in 10 organisms) and *Cell motility *(enriched in 9 organisms), while cell cycle functions were enriched in six species, belonging to cluster A, including *Caulobacter *and *Neorickettsia sennetsu *of cluster F. These results confirmed experimental data on the functions controlled by CtrA (see Background section) in *Caulobacter *suggesting that (i) the analysis of the regulon is able to capture good candidates of CtrA targets and (ii) the control of CtrA over these functions is at least partially evolutionarily conserved.

### CtrA-DnaA-GcrA-CcrM connections

In *Caulobacter*, transcriptional regulation of *ctrA *is based on a positive feedback loop that includes CtrA itself, GcrA, DnaA and CcrM.

As reported in Figure S3 (Additional file 9) (see also Additional file 7, Table S4) the presence of CcrM methylation sites upstream of cell cycle related genes was assessed (see Methods section). For this analysis we used the consensus methylation site of CcrM, i.e. GANTC [47]. As reported elsewhere [39,48], methylation by CcrM is conserved among alphas and *ccrM *genes from *Caulobacter *and *S. meliloti *which are able to cross-complement deletions, suggesting that the methylation site might be conserved. In a homogeneous background of DNA, the expected frequency of this sequence is 4/1024 nucleotides, i.e. two occurrences in the 500 bp long window that was used to define the promoter region for motif finding. The average number of occurrences found ranged from 0.4 to 1.5 methylation sites per promoter (defined as the 500 bp from the first 100 bp of a gene to 400 bp upstream of the translation start site), according to non-uniform distributions of nucleotides in these genomes. Although some genes possess more predicted methylation sites in their promoter region, it is not possible to derive a possible control of methylation in their expression.

**Figure 5:**
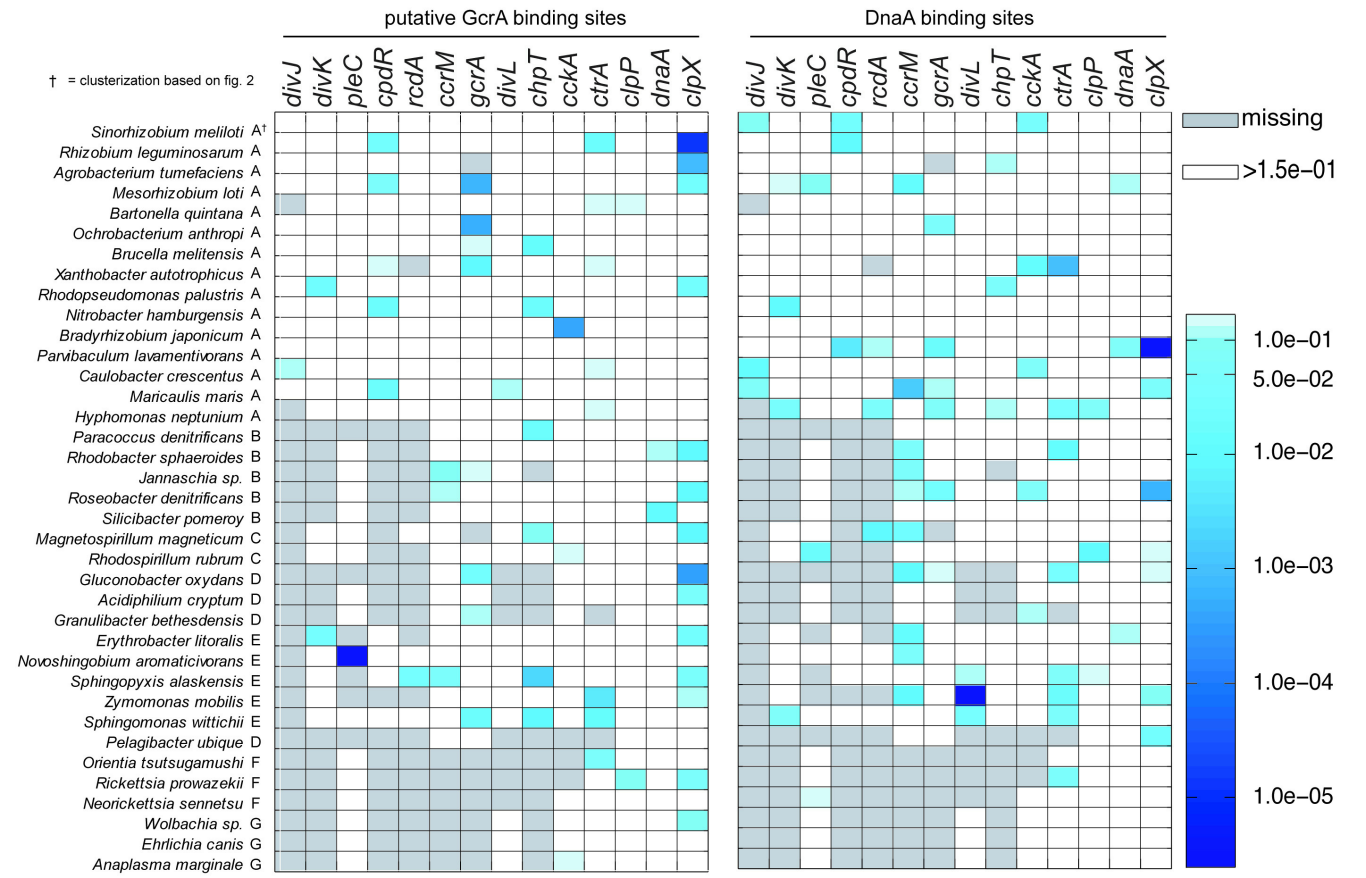
**Transcriptional control of GcrA and DnaA on cell cycle genes**. GcrA and DnaA binding sites on factors involved in cell cycle regulation among alpha-proteobacteria (see also Additional file 7, Table S4).

The consensus site of DnaA is very conserved among bacteria, in fact, DnaA experimental binding sites in *E. coli *and *B. subtilis *differ only by a nucleotide [49,50] which corresponds to that found in *Caulobacter*, supporting the conclusion that its binding site is conserved across alphas. Promoter regions of orthologs in all alphas were also scanned using the DnaA matrix based on 15 known DnaA motifs in *Caulobacter *(see Methods section) and results are shown in Figure 5 (see also Additional file 7, Table S4). Again, is the DnaA binding site conserved across the alpha proteobacteria? Following this analysis, the promoter sequence of *gcrA *does not bear a significant DnaA motif in *Caulobacter*, while elsewhere the control of DnaA on the *gcrA *promoter has been proposed [51]. It is worth noting that a predicted DnaA motif is present upstream of the *gcrA *gene from two closely related species *Hyphomonas neptunium *and *Maricaulis maris*. Moreover, in *Caulobacter*, the presence of DnaA binding sites was observed upstream of *divJ *and *cckA*, targets that are not confirmed by previous experimental analysis. As in the case of GcrA, the absence of conservation of putative binding sites at the taxonomic level was observed, with the only exception being the suggested DnaA control on CtrA in several species. This might be caused by the low specificity of the DnaA and GcrA matrices used for motif finding, but may also suggest that in some alpha-proteobacteria the DnaA->GcrA->CtrA circuit may be simplified by excluding GcrA. The control of DnaA on CcrM is also interesting because it mainly concerns those organisms where *ccrM *lacks CtrA binding sites. The opposite is also true: *ccrM *is very often preceded by CtrA binding sites in Rhizobia and it lacks DnaA motifs. This questions the existence of a DnaA->CcrM->CtrA circuit in Rhizobia and suggests an at least partial decoupling of CtrA activity (modulated through control of the *divJ*-*pleC-divK *system, which acts on the phosphorelay) from DNA replication triggered by DnaA. Otherwise, the absence of CtrA binding sites upstream of DnaA might suggest the existence of other not yet identified regulators, which may connect CtrA and DnaA in these organisms.

Although GcrA is considered a DNA binding protein activating transcription, no experimental evidence has ever been proposed to demonstrate this behavior [19]. However it is still possible that DNA sequences are associated with the presence of this factor. GcrA putative binding sites were searched for in promoter regions of genes encoding factors involved in cell cycle regulation using a strategy similar to the one followed to predict CtrA regulons (see Methods section). In *C. crescentus*, as reported in Figure 5 (see also Additional file 7, Table S4), the existence of a putative GcrA binding motif (Additional file 10, Figure S4) upstream of *ctrA *and also the presence of such motifs upstream of *divJ *was confirmed. Concerning the other species, patterns of occurrence did not respect phylogenetic relationships. The only gene for which most of the organisms seem to possess a GcrA binding site is *clpX*.

### Verification of binding site prediction

**Figure 6:**
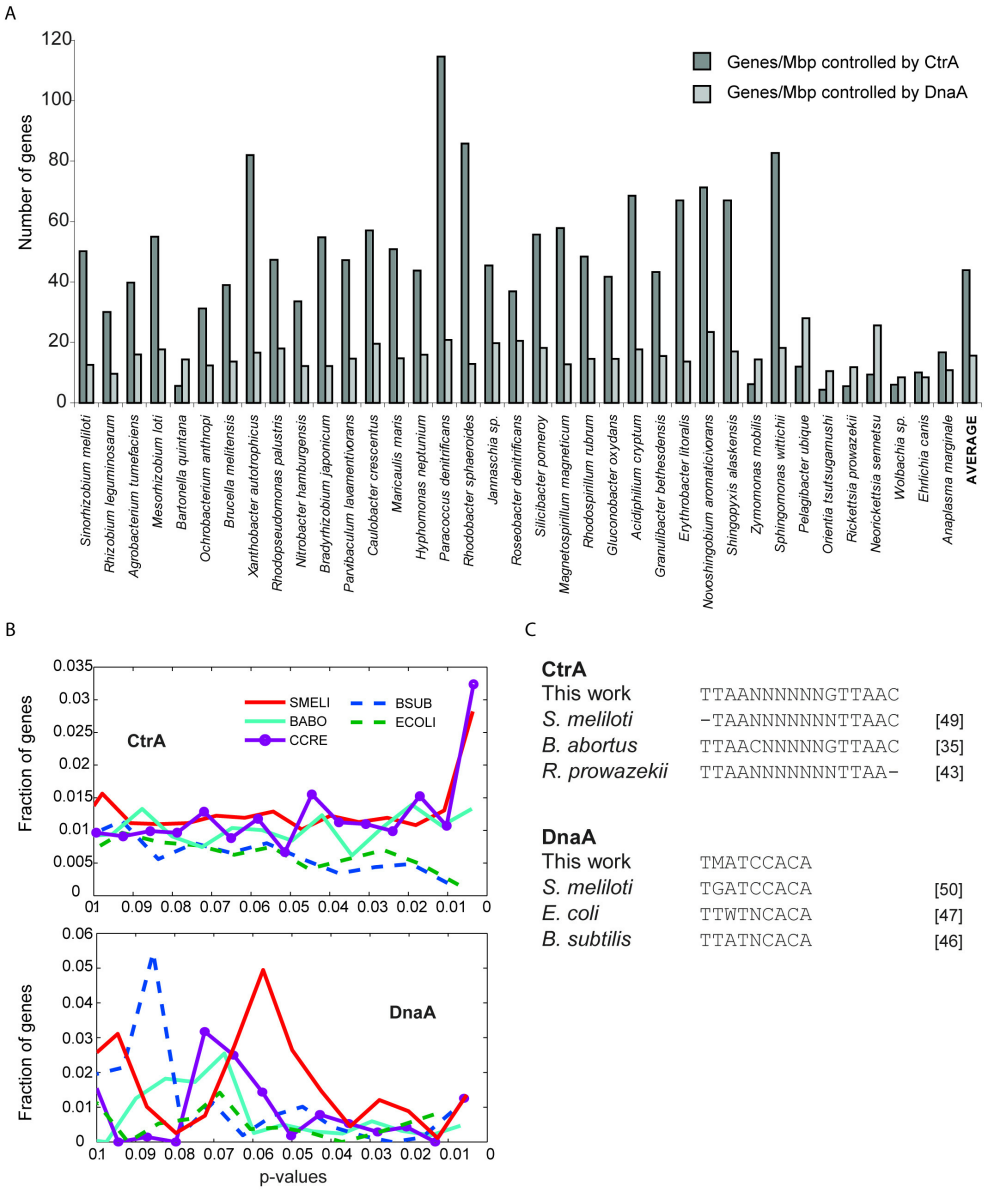
**Verification of CtrA and DnaA binding site prediction**. A. Number of genes putatively controlled by CtrA (dark gray) and DnaA (light gray) across alpha-proteobacteria. B. Distribution of p-values assigned to each gene with respect to the CtrA binding and DnaA binding for three alpha-proteobacteria (CCRE = *C. crescentus*, BABO = *B*. *abortus*, SMELI = *S. meliloti*) and species, possessing only DnaA (ECOLI = *E*. *coli*, BSUB = *B*. *subtilis*). The distributions of CCRE, BABO, SMELI for CtrA start to diverge from the 'background' distributions represented by organisms not possessing CtrA at a p < 0.05, while for DnaA this distribution is uniform among all five organisms. C. Consensus sequences of DnaA and CtrA in different bacteria found experimentally elsewhere (see references near the sequences) compared with our PMWs. M = T or G, W = A or T.

The prediction of CtrA and DnaA binding sites across alphas is based on *Caulobacter *data and we have already discussed how, from previous studies, it is possible to hypothesize that CtrA and DnaA (and also CcrM) binding sites are conserved across the alpha-proteobacteria. However our prediction ability might be accurate only for bacteria closely related to *Caulobacter*, but, going farther, this confidence could decrease. To evaluate this bias in binding site prediction, we counted the number of genes in each genome putatively controlled by CtrA and DnaA, normalized for the genome size. We found (Figure 6A) that the number of predicted genes is fairly constant and depends only on the genome size (or number of genes), suggesting that our prediction confidence is not biased by the phylogenetic distance. This result also explains the success of the complementations of *ctrA *deletion in *Caulobacter *by orthologs from other alpha proteobacteria, as discussed in the previous sections [46,52]. We also evaluated whether the presence of CtrA and DnaA predicted genes depended on the presence of CtrA and DnaA themselves in the genomes or if it was an artifact of bioinformatic analysis. We therefore plotted the fraction of genes controlled by CtrA and DnaA at small p-values in three alpha proteobacteria possessing CtrA and DnaA and in *E. coli *and *B. subtilis*, which possess only DnaA (Figure 6B). From this analysis it is evident that, at lower p-values, only organisms with CtrA keep a consistent fraction of genes controlled by CtrA, while for DnaA, which is present and active in all, every organism maintains a similar fraction of putatively controlled genes--even at lower p-values.

CtrA binding site consensus has been previously tested experimentally in *R. prowazekii, S. meliloti *and *B. abortus*, besides *Caulobacter *[38,46,52]. Here, we compared the experimental consensus sequences with our bioinformatic PWM (Figure 6C), and our prediction coincides with experimentally identified sequences. Our PMW corresponds also with a CtrA PMW previously found [53].

The DnaA binding site has been studied in very diverse bacteria such as Gram-negative *Escherichia coli *and Gram-positive *Bacillus subtilis *[49,50]. The DnaA binding site in these two species differs because of one nucleotide, suggesting that the binding site should also be very conserved in alpha proteobacteria. We compared the predicted PWM for DnaA based on *C. crescentus *with experimental DnaA binding sites in *E. coli*, *B. subtilis *and *S. meliloti *(Figure 6C) [49,50,54]. Our prediction, based on nucleotide sequences that bind DnaA in *Caulobacter*, corresponds to binding sites experimentally found in other bacteria.

This verification was possible only for DnaA and CtrA, while GcrA has been studied only in *C. crescentus *and experimental data are available only in this organism. It has not been clarified whether GcrA binds DNA directly or through an unknown factor X [19]. Therefore, since the knowledge on GcrA is still preliminary and experimental work still needs to be done, we limited the experimental validation to CtrA and DnaA, for which data are available. It should be noted, however, that both DnaA and CtrA experimental verifications revealed that our method is accurate and reliable.

### Reconstruction of regulatory circuits

**Figure 7:**
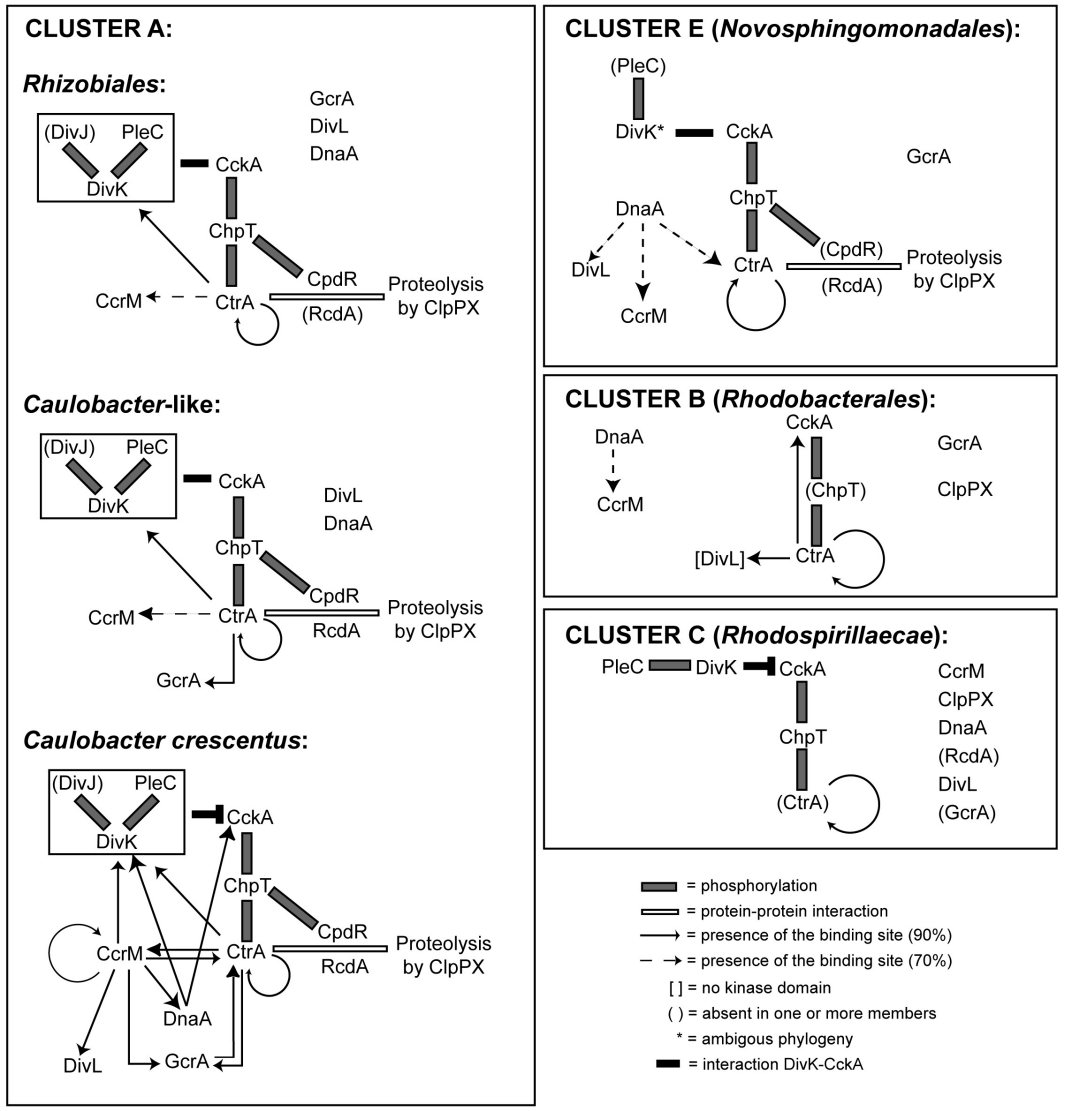
**Regulatory circuits of clusters A, B, C, E**. See the "Results" and "Discussion" sections for more details. Interactions via phosphorylation, as well as proteolysis, were suggested only considering the interaction demonstrated in *Caulobacter*. Moreover, DivK inhibition on CckA was considered, as in Biondi (2006), only in *Caulobacter *[20]. The presence of binding sites of transcription factors CtrA, DnaA and GcrA is shown as a continuous line if predicted binding sites were present in at least 90% of the gene promoters of a cluster. In contrast, the connection is shown as a dotted line for binding sites present in ca. 70%. The *Caulobacter*-like group corresponds to *B. japonicum*, *P. lavamentivorans *and *M. maris*.

Based on data of Figures 2, 3, and 5, we reconstructed the architecture of the seven clusters (A to G) found in the BBH analysis; as discussed below, only four clusters revealed a defined architecture as illustrated in Figure 7. Models of CtrA regulation are shown in the clusters (clusters A, B, C and E) where interactions between factors were found. This modeling is essential in order to underline differences and conservation of several features of cell cycle regulation in alpha proteobacteria.

Cluster A (*Caulobacterales*, *Rhizobiales *and several *Rhodobacterales*) contains the larger number of genomes analyzed here and the organization of cell cycle genes resembles that observed in *Caulobacter *(see bottom part of Figure 7 for details), i.e. it includes the phosphorelay CckA-ChpT-CtrA/CpdR and also the proteolysis machinery composed by the ubiquitous ClpPX protease, CpdR and RcdA, which is however absent in *Xanthobacter*. The DivJ-PleC-DivK system is conserved and corresponding genes are controlled by CtrA in all members of the cluster. CcrM also controls CtrA and GcrA.

*Rhizobiales *are different from bacteria similar to *Caulobacter *(*B. japonicum*, *P. lavamentivorans *and *M. maris*) due to the absence of the CtrA control on GcrA which is present only in the *Caulobacter-*like.

In cluster E, the CckA-ChpT-CtrA phosphorelay is present with the second branch also leading to phosphorylation of CpdR that, together with RcdA, are thought to be involved in controlling CtrA proteolysis. DivK is absent in this cluster although a *divK*-like gene has been found although it has an anomalous phylogeny (Additional file 3, Figure S1). In most members of cluster B, CtrA controls its own promoter, other genes involved in cell division and chromosome partitioning as well as *ccrM*.

In cluster B, the CckA-ChpT-CtrA regulon is isolated from GcrA, CcrM and DnaA while CtrA controls itself as well as two factors involved in its phosphorylation, CckA and DivL.

In this group, DivL lacks the kinase domain that is usually present only when DivK is also present (data not shown, based on SMART database). In fact, DivK is absent in cluster B together with its kinase/phosphatase. The fact that CtrA controls its kinase creates theoretically a feedback.

In cluster C, the CckA-ChpT-CtrA phosphorelay is present while CpdR is absent. Also CtrA is not connected with DnaA and CcrM and finally DnaA has binding sites on *ccrM *and *divL*. Connections between DnaA/CcrM and CtrA seem to be achieved by the PleC-DivK two-component system. No clear positive or negative transcriptional feedbacks of CtrA on other cell cycle factors are present.

Cluster D contains the two cases among alphas, *Granulibacter *and *Pelagibacter*, where a *ctrA *ortholog has not been found. The phosphorelay, even in organisms of cluster E that have CtrA, is degenerated; although *cckA *is present, no orthologous gene of *chpT *has been found. There is no explanation for the presence of CckA in organisms with no CtrA. Since histidine phosphotransferases are difficult to annotate [10,20], it is possible that other phosphotransferases substitute for ChpT in those organisms containing both CtrA and CckA, as proposed here for cluster G.

## Discussion

The cell cycle 'engine' (controlling DNA replication, cell division, morphogenesis of polar structures) is an essential machine in every living organism, and a major goal of molecular cell biologists is to uncover how this engine works in every organism. We have performed a bioinformatic analysis of cell cycle control genes in the alpha proteobacteria, taking cues from the well-characterized control system in *Caulobacter *(see Background section for details).

The procedure used here is able to detect only loss of regulatory points with respect to *Caulobacter*. However, the knowledge of conservations and variations from the *Caulobacter *scheme can be extremely useful for future studies of the cell cycle network in all alpha-proteobacteria.

### Conservation and variability of the CtrA regulatory system

CtrA regulation is thought to play an essential role in most alpha-proteobacteria cell cycle progression while its activity varies during each cell cycle in response to several levels of control in *Caulobacter*. As illustrated in Figure 7, many regulators are connected with CtrA, via epigenetic, transcriptional and post-translational mechanisms in most of the clusters. The reconstructed architecture within alpha-proteobacteria of regulatory pathways that are involved in controlling CtrA activity is however surprisingly variable, suggesting diverse evolutionary pathways in each cluster.

CtrA, even if associated with variable regulatory circuits within alphas, shows conservation of the control of certain functions (Figure 4) such as cell division, motility and signal transduction, especially in cluster A where CtrA is, in fact, the master regulator of cell cycle. The ability to detect the same functions controlled by CtrA in different organisms also suggests that the prediction capability of CtrA-regulated genes is highly reliable.

The *C. crescentus *regulatory scheme of CtrA in other members of cluster A shows several variations, especially in *Rhizobiales*. For example, the control of CtrA on the response regulator *divK*, observed in *C. crescentus*, is shifted to the gene encoding the DivK kinase (*divJ*) and/or the phosphatase (*pleC*) in most *Rhizobiales*. This observation may suggest that feedbacks can be conserved even when connections are rewired.

Another interesting feature revealed by this study is the control of CtrA on *cckA *and *divL *in several members of the *Rhodobacterales *(cluster B), which is coupled to the absence of the system regulating CckA activity (the DivK/DivJ/PleC two-component system). Thus, in cluster A members, CtrA controls the *divK-divJ-pleC *transcription and DivK presumably inhibits CckA activation that is indeed able to activate CtrA through a phosphorylation cascade, while in cluster B *Rhodobacterales *our results suggest that CtrA acts directly on *cckA *transcription and thus it may directly modulate the CckA quantity in the cytoplasm.

#### Other regulatory circuits (DivL-DivK and DnaA-GcrA-CtrA)

Several conclusions can be made at the molecular level from this bioinformatic approach on specific factors such as DivL-DivK or the DnaA-GcrA-CtrA system. DivL, that still lacks a precise function, and DivK, have been connected in previous studies since DivL was detected in a yeast-two hybrid experiment using DivK as bait [55]. Alleles of *divL *were also able to complement *divK *defective alleles [24,56]. A further association between DivL and DivK was found in our study whereby orthologs of DivL can be found in alpha-proteobacteria either as complete kinases (such as in *Caulobacter*) or without the kinase domain (data not shown); this latter form is always associated with the absence of DivK--suggesting that the kinase domain of DivL functions together with DivK. This observation is also consistent with previous studies that showed that DivL interacts with DivK using the kinase domain and has two separate functions in *Caulobacter *[56]. The observation that in several alphas DivL keeps only the sensor part is also paralleled by previous studies showing that its kinase activity in *Caulobacter *is dispensable [56].

It can be concluded from this study that the DnaA-GcrA-CtrA regulatory system in *Caulobacter *is not conserved throughout alpha-proteobacteria. It is however true that DnaA controls *gcrA *in several members of cluster A (surprisingly this control in *Caulobacter *was not detected) and that GcrA controls *ctrA *in few alphas, including in *Caulobacter*. This observation might suggest that this important loop in the regulation of the *Caulobacter *cell cycle is instead dispensable in other bacteria of cluster A. This conclusion is also consistent with the result that regulation of *ctrA *transcription can be removed without affecting *Caulobacter *viability although robustness of the system might be compromised.

### Evolution of alpha-proteobacteria cell cycle

Finally, the reconstruction of the evolution of the regulatory schemes found was undertaken following phylogeny proposed elsewhere [42,57]. *Rickettsiales *(clusters F and G) were excluded from this model due to their massive genome reduction that has been previously shown to be a consequence of the evolution of an obligate intracellular life in eukaryotic cells [58].

The regulation of cell cycle progression in *Caulobacter *has evolved in order to respond to a lifestyle in nutrient-poor environments but other alpha-proteobacteria occupy different ecological niches, suggesting that cell cycle regulation must respond to different requirements; from an evolutionary perspective this means that features found in *Caulobacter *should not be completely conserved in other alpha-proteobacteria, especially those experiencing different environments. Conversely, similarities between closer organisms were expected due to common phylogenetic ancestries.

Gupta and Mok [42] proposed that *Rhodospirillales *and *Novosphingomonadales *(clusters C, D and E) branched earlier than the other alphas (clusters A and B), and after *Rickettsiales *(clusters F and G). Two of those "older" clusters (C and E) show, in fact, a different arrangement of regulatory factors although none show circularity of the regulation created by feedbacks. From this primordial situation, when alphas were experimenting different options, two situations seem to have evolved: a complex circuit (cluster A) and a minimal organization (cluster B). In both clusters two interlaced oscillators are present: besides cluster A where this organization has already been proposed [20], cluster B, also shows a CtrA auto-regulatory circuit and a CtrA-CckA-ChpT-CtrA oscillator. It is also interesting to point out that the minimal circuit of cluster C corresponds to a situation where CtrA is not essential.

## Conclusions

This is the first systematic attempt to translate the information accumulated over the years on cell cycle regulation in the model *Caulobacter *system into a common body of knowledge regarding the whole taxonomic group of alpha-proteobacteria. Results suggest that the scheme found in *Caulobacter *may work in closely related bacteria such as those belonging to *Caulobacterales *and *Rhizobiales *while in other alphas this conservation is lost even though several modules are present. Finally, this analysis represents an important step for all future cell cycle studies in alpha-proteobacteria, offering many experimental scenarios designed to confirm or reject the observations made here.

## Methods

### Phylogenetic Tree

To construct our reference phylogenetic tree, from the server of the RDP (ribosomal database project) [59], we downloaded the *E. coli *sequences corresponding to universal proteins in prokaryotes: FusA, IleS, LepA, LeuS, PyrG, RecA, RecG and RplB. Then we blasted these sequences against alpha-proteobacterial genomes chosen for analysis, retrieving the BBH for each protein in each genome. Each dataset was aligned using Muscle [60] and the obtained multi-alignments were then joined head-to-tail in a single concatamer of 5000 sites. This alignment was used with the software Mega 4 [61] running the Neighbor-Joining algorithm, 500 bootstrap replicates and the Dayhoff model of evolution.

### Ortholog Identification

We used a dataset comprising all completely sequenced genomes of organisms belonging to the alpha-proteobacteria for ortholog identification (Additional file 1, Table S1). Orthologous genes were identified with the so-called bidirectional best hit (BBH) criterion: the relation of gene *x *in genome *A *and gene *y *in genome *B *is called bidirectional best hit, when *x *is the best hit of query *y *against all genes in *A *and vice versa. We used two different datasets of queries coming from *C. crescentus *and *B. melitensis *and we also performed a phylogenetic analysis of those proteins found using the BBH analysis; the *Caulobacter *dataset is derived from experimental data [13] while that from *B. abortus *is derived from both experimental [38] and bioinformatic analysis performed using *Caulobacter *genes. For multi-domain proteins we used separated domains and discarded highly variable regions from the analysis. The two sets of BBH sequences orthologous to *Caulobacter *and *Brucella *sequences were then combined to collect the maximum number of proteins. When *C. crescentus *and *B*.*melitensis *sequences retrieved two different proteins for the same gene, we collected both of them and used phylogenetic methods to solve the mismatch; when one of the two queries returned no BBH we used only the one available. We used a 0.0001 e-value threshold in the Blast analysis.

### Regulons of CtrA, GcrA, DnaA and CcrM methylation sites characterization

Genes directly regulated by CtrA in alpha-proteobacteria genomes were identified using the following multilevel approach:

1) A Position Weight Matrix (PWM) describing CtrA binding sites in *C. crescentus *was obtained by using the AlignAce program [45] on upstream sequences of 54 genes previously identified as being part of the CtrA regulon [13]. We used such a matrix to scan all genomes considered in this work (see Additional File 1, Table S1 for all organisms list) with a sliding window approach and a scoring function from Schneider and collaborators (1986) [62]: S_i _= (1/L) Σ_j _[2 + log_2_(F_ij_)], where F_ij _is the frequency of base i at position j of the L-mer. This score, whose maximum for the best match using a CtrA position weight matrix is ≈1.22, is a measure of the information content of a potential binding site.

2) We retained only motifs having a score ≥30% of the maximum score attainable with the given matrix and located in the range -100 to 400 nucleotides from the start codon of a gene.

3) Then we applied a Z-score transformation to highlight significant occurrences and take into account the background DNA implicitly: Z_i _= (S_i _- <S_i_>)/σ_i_, where S_i _is calculated using the above formula, and <S_i_> is the average score in an organism over all L-mers and σ_i _is the corresponding standard deviation.

For GcrA and DnaA the strategy followed was the same, except that PWMs were obtained differently. For GcrA we obtained a PWM describing a motif enriched upstream of sequences controlled by GcrA as proposed by others [19]. By using microarray and GcrA deficient strains, a list of genes whose expression changes significantly when GcrA is mutated was obtained. We used the 48 genes with maximum fold change in that list. The corresponding upstream sequences were retrieved and analyzed with AlignAce [45] and MDscan [63]. In both cases a high scoring motif (16 bp, see Additional file 10, Figure S4) present in a high percentage of the input sequences was obtained, and was used for subsequent scanning of the genomes of our dataset (Figure 5 and Additional file 7, Table S4).

DnaA recognizes a sequence whose consensus in *Caulobacter *is [TC] [TCG] [AG]TCCACA as previously reported [18]. In the same work, 15 DnaA binding sites were shown and were used to build a position weight matrix specific for DnaA (Additional file 10, Figure S4). However AlignAce and MDscan were not able to identify motifs for DnaA corresponding at least partially to the proposed consensus using two datasets of genes previously proposed [18].

Both GcrA and DnaA were then analyzed as reported in points 2 and 3 for CtrA. CcrM methylation targets were searched using a regular expression modelling the known target of this methyl-transferase (GANTC) [11].

### Functional Enrichment

We took advantage of COG [64] categories to evaluate the functional enrichment of the genes identified with the approach described above. Supposing that the regulon dataset of a genome is composed of N genes, we counted the number of genes for each category and then repeated the same count for 10,000 N-sized groups of genes randomly picked from the genome. We obtain a p-value describing functional enrichment in the original dataset by counting how many times a COG category is more represented in the random group and then dividing it by the total number of repetitions performed.

## Abbreviations

PWM: position weight matrix; BBH: bidirectional best hit; COG: clusters of orthologous groups of proteins; SMART: simple modular architecture research tool

## Authors' contributions

EGB planned the project; MB performed the analysis; MF, RF, AM, LF and MB helped with the analytical work and the writing process. EGB and MB wrote the manuscript and created Tables and Figures. All authors read and approved the final version of the manuscript.

## Supplementary Material

Additional file 1 **Table S1**. The list of the 65 organisms used in this work. Click here for file

Additional file 2 **Detailed description of additional material**. Legends of additional Tables and Figures. Click here for file

Additional file 3 **Figure S1**. Phylogenetic profiling of BBH hits in the 65-genome dataset. Click here for file

Additional file 4 **Table S2**. Bidirectional Best Hits. Click here for file

Additional file 5 **Figure S2**. Phylogenetic trees of cell cycle related proteins. Click here for file

Additional file 6 **Table S3**. CtrA, DnaA, GcrA and CcrM binding sites. Click here for file

Additional file 7 **Table S4**. Numerical values for Figures 3, 5 and S3. Click here for file

Additional file 8 **Table S5**. Numerical values for Figure 4. Click here for file

Additional file 9 **Figure S3**. Control of CcrM on cell cycle genes. Click here for file

Additional file 10 **Figure S4**. Sequence logo of the putative GcrA motif. Click here for file
