# Supplementary material for: The diversity and evolution of cell cycle regulation in alpha-proteobacteria: a comparative genomic analysis
Source: BMC Syst Biol. 2010 Apr 28;4:52. doi: 10.1186/1752-0509-4-52 (PMC2877005; doi:10.1186/1752-0509-4-52)
Supplement: Additional file 2 — Detailed description of additional material. Legends of additional Tables and Figures. [file 1752-0509-4-52-S2.PDF]

Additional files

The diversity and evolution of cell cycle regulation in alpha-proteobacteria: a comparative genomic analysis

Brilli M, Fondi M, Fani R, Mengoni A, Ferri L, Bazzicalupo M, Biondi EG

LEGENDS:

Figure S1 – Phylogenetic trees of cell cycle related proteins.

Orthologous sequences retrieved using the BBH method (see Material and Methods) were aligned using Muscle (Edgar, 2004) with 10^5 iterations; alignments were manually refined to eliminate badly aligned regions and the Mega program was used to build Neighbor-joining trees. The evolutionary model for each tree was the Dayhoff one (Schwarz & Dayhoff, 1979).

Figure S2 – Phylogenetic profiling of BBH hits in the 65-genome dataset.

The same as Figure 2, for all genomes considered (listed in Table S1).

Fig. S3 Control of CcrM on cell cyclegenes.

CcrM methylation sites on genes encoding for factors that control cell cycleprogression in *Caulobacter* (see also table S4).

Figure S4 – Sequence logo of the putative GcrA motif.

We analyzed the 500 upstream nucleotides of 50 *C. crescentus* genes identified by Holzendorff et al. (2004) as putative direct targets of GcrA using AlignAce (Roth et al., 1998). The top scoring motif was then retrieved from the 50 sequences and aligned to obtain the sequence logo shown in this figure (Crooks et al., 2004). The sequence logo consists of stacks of symbols, one for each position in the motif. The height of the stack indicates the sequence conservation at that position in bit units (max value attainable with DNA $\log_2 4 = 2$ bits), while the height of symbols within each stack corresponds to the relative frequency of each base at that position.

Table S1 – The list of the 65 organisms used in this work.

A reduced, non-redundant dataset comprising 37 organisms was used for the analysis of transcriptional regulation.

Table S2 – Bidirectional Best Hits.

A list of the GI identification number of each identified BBH hit from the 65 genomes.

Table S3 – CtrA, DnaA, GcrA and CcrM binding sites

Position weight matrices for the regulators analyzed in this work.

Table S4 – Numerical values for figures 3, 5 and S3

Z-scores (see Methods section for details) for figures 3, 5 and CcrM binding sites for figure S3.

Table S5 – Numerical values for figure 4

The calculation of p-values of functional enrichment based on COG categories and results from the CtrA regulon prediction. A gene has been considered here as being controlled by CtrA if the Z-score of its upstream region for the CtrA motif is higher than 2.

References

1. Crooks GE, Hon G, Chandonia JM, Brenner SE WebLogo: A sequence logo generator, Genome Research, 14:1188-1190, (2004)
2. Edgar, Robert C. (2004), MUSCLE: multiple sequence alignment with high accuracy and high throughput, Nucleic Acids Research 32(5), 1792-97.
3. Holtzendorff J, Hung D, Brende P, Reisenauer A, Viollier PH, McAdams HH, Shapiro L: Oscillating global regulators control the genetic circuit driving a bacterial cell cycle. Science 2004, 304(5673):983-987.
4. Laub, M.T., Chen, S.L., Shapiro, L. and McAdams, H.H. (2002) Genes directly controlled by CtrA, a master regulator of the Caulobacter cell cycle. Proc Natl Acad Sci U S A, 99, 4632-4637.
5. Roth FP, Hughes JD, Estep PW, Church GM: Finding DNA regulatory motifs within unaligned noncoding sequences clustered by whole-genome mRNA quantitation. Nat Biotechnol 1998, 16(10):939-945.
6. Schwarz R & Dayhoff M (1979) Matrices for detecting distant relationships. In Dayhoff M, editor, Atlas of protein sequences, pages 353 - 58. National Biomedical Research Foundation.
