# Supplementary figures and images for: The diversity and evolution of cell cycle regulation in alpha-proteobacteria: a comparative genomic analysis
Source: BMC Syst Biol. 2010 Apr 28;4:52. doi: 10.1186/1752-0509-4-52 (PMC2877005; doi:10.1186/1752-0509-4-52)

Figure S1

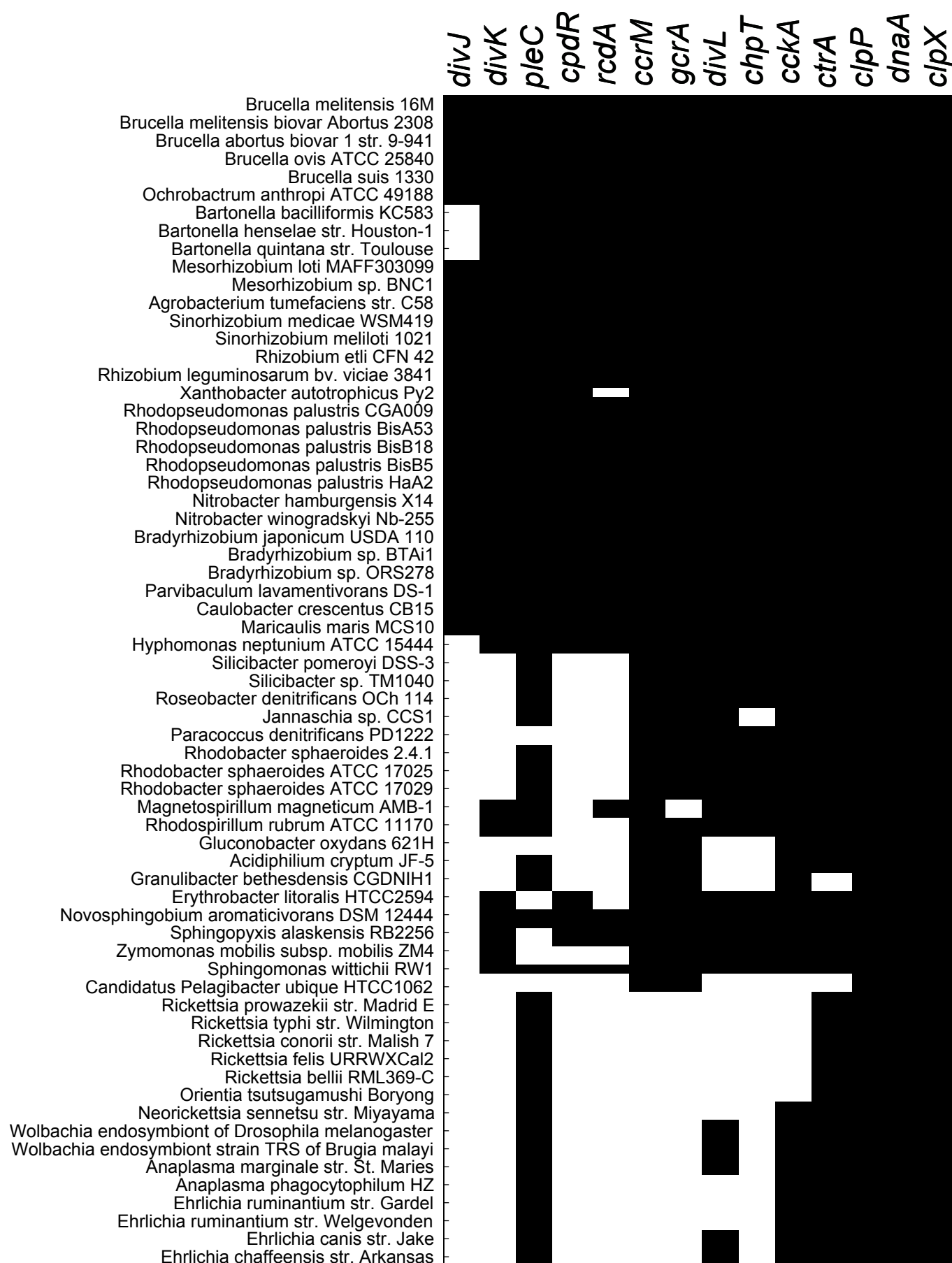

Supplement: Additional file 3 — Figure S1. Phylogenetic profiling of BBH hits in the 65-genome dataset. [file 1752-0509-4-52-S3.PDF]

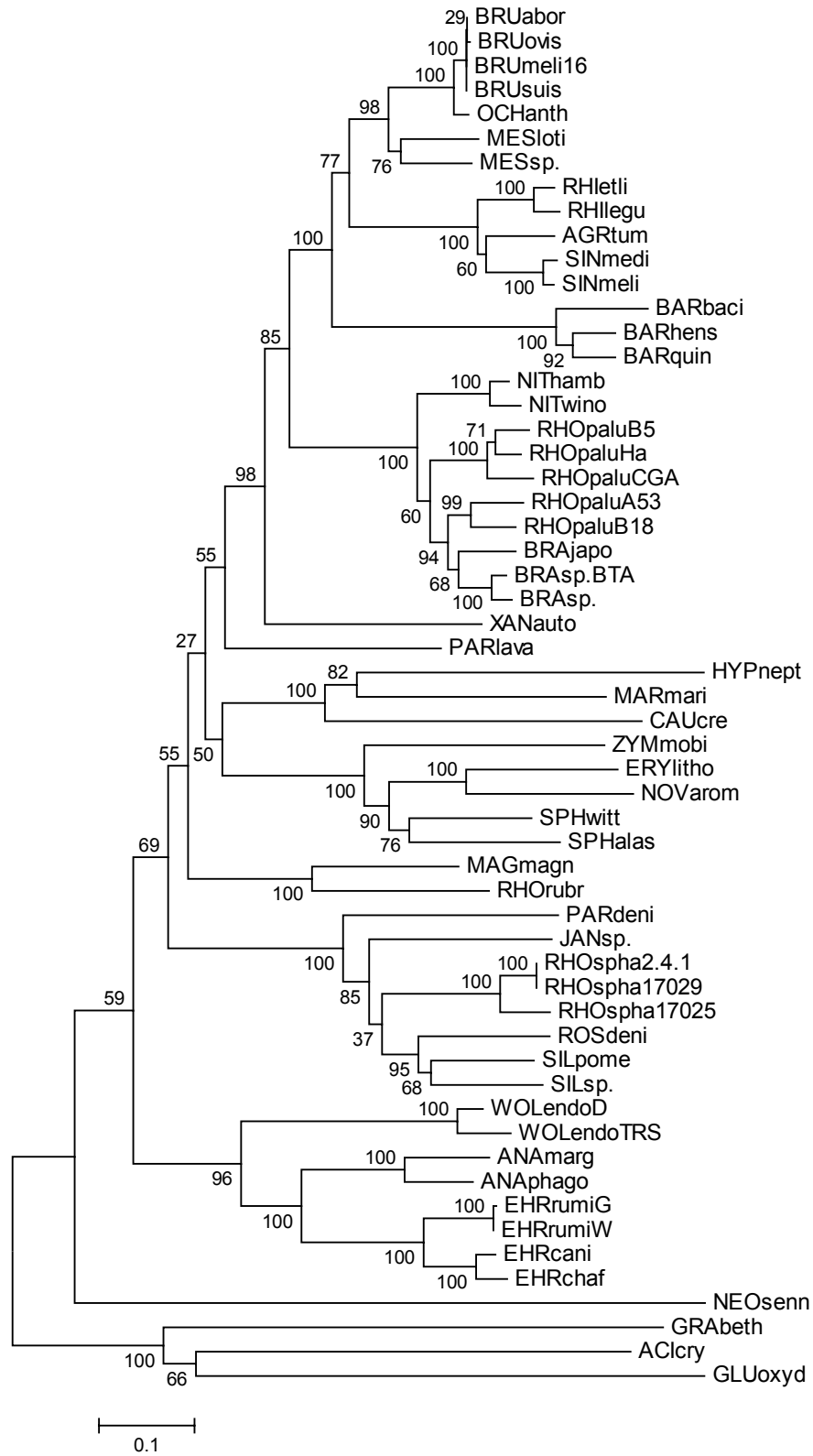

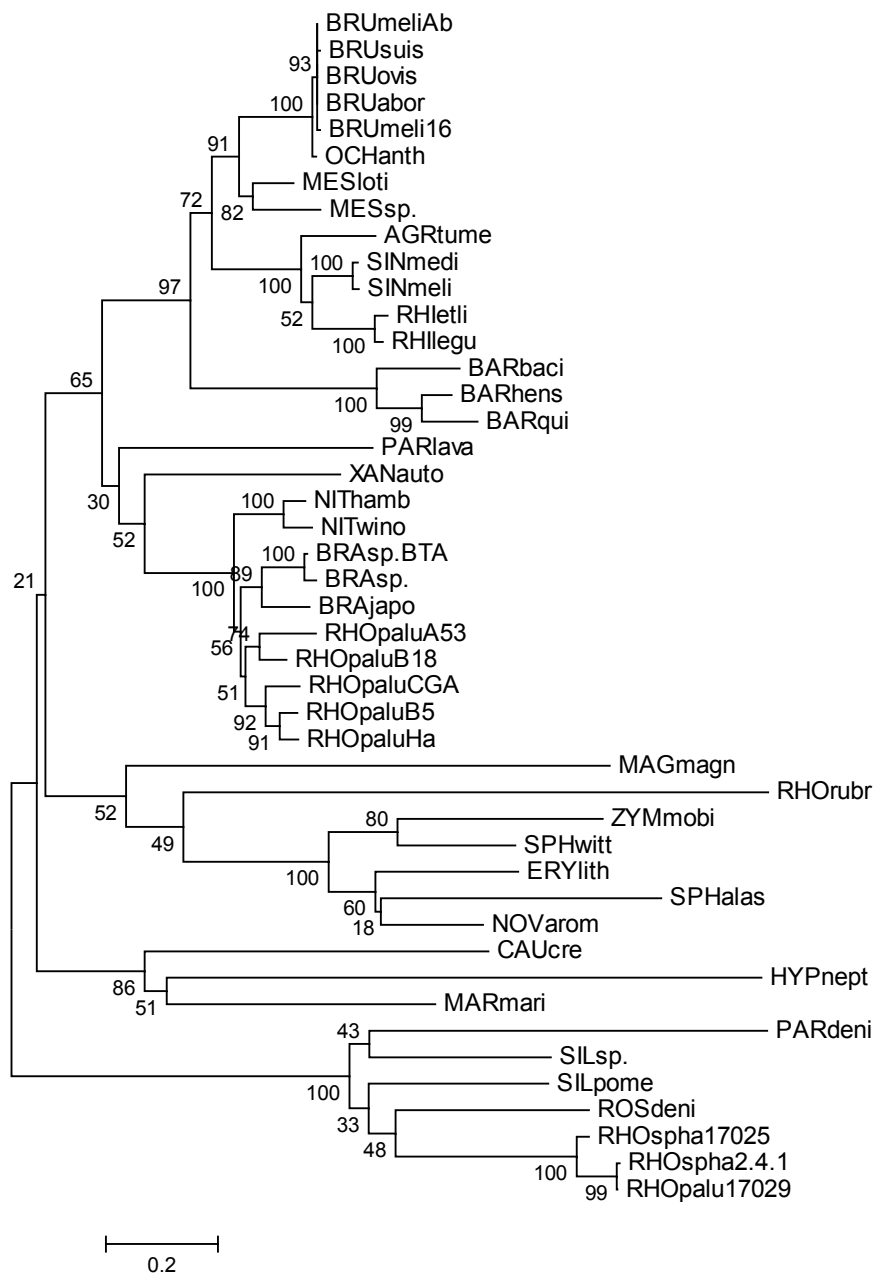

DivJ

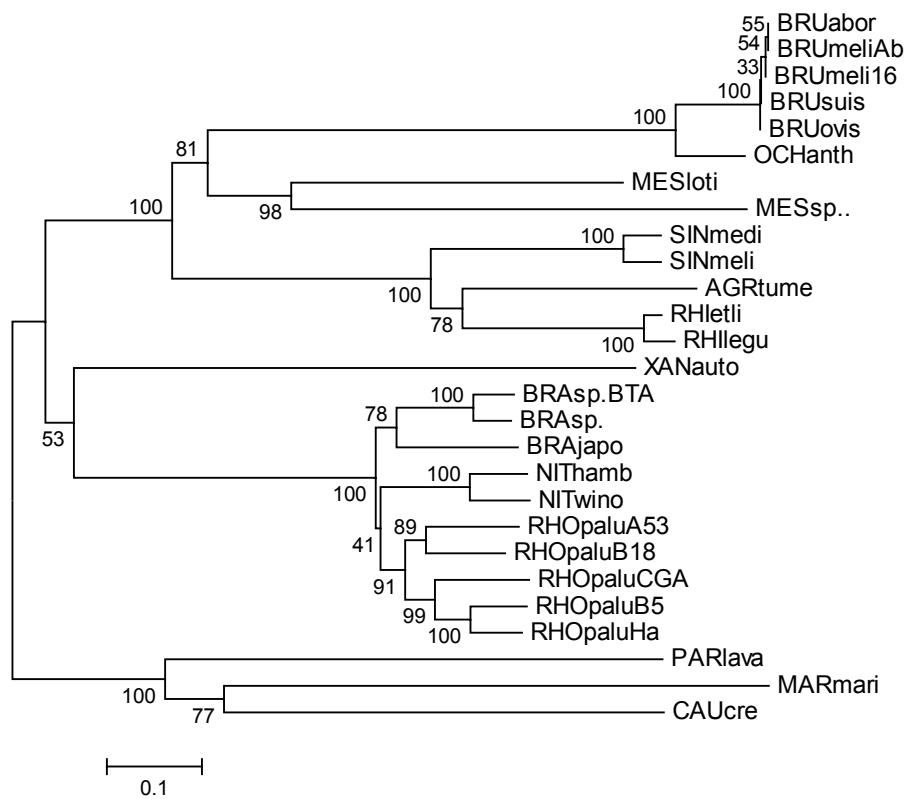

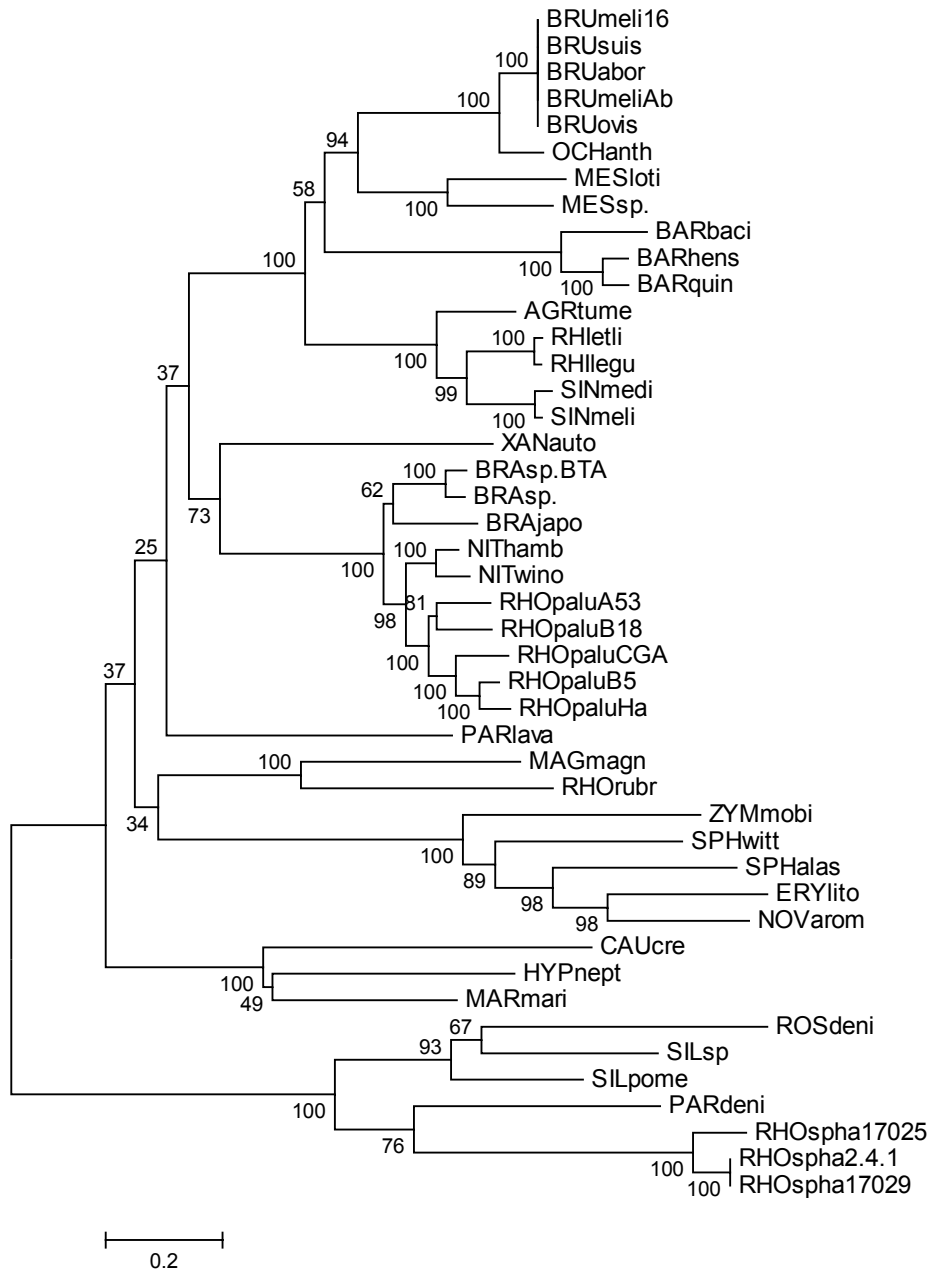

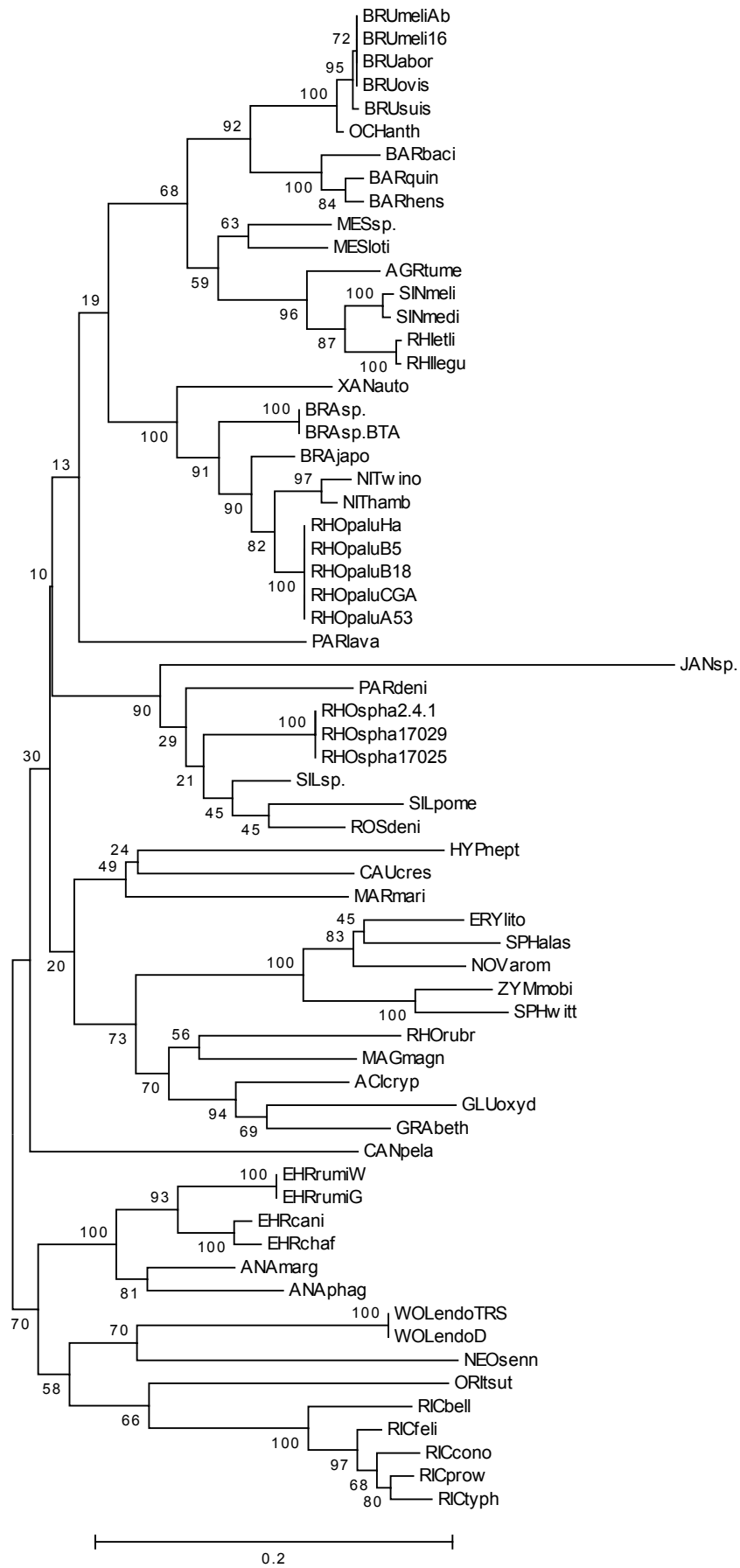

RcdA

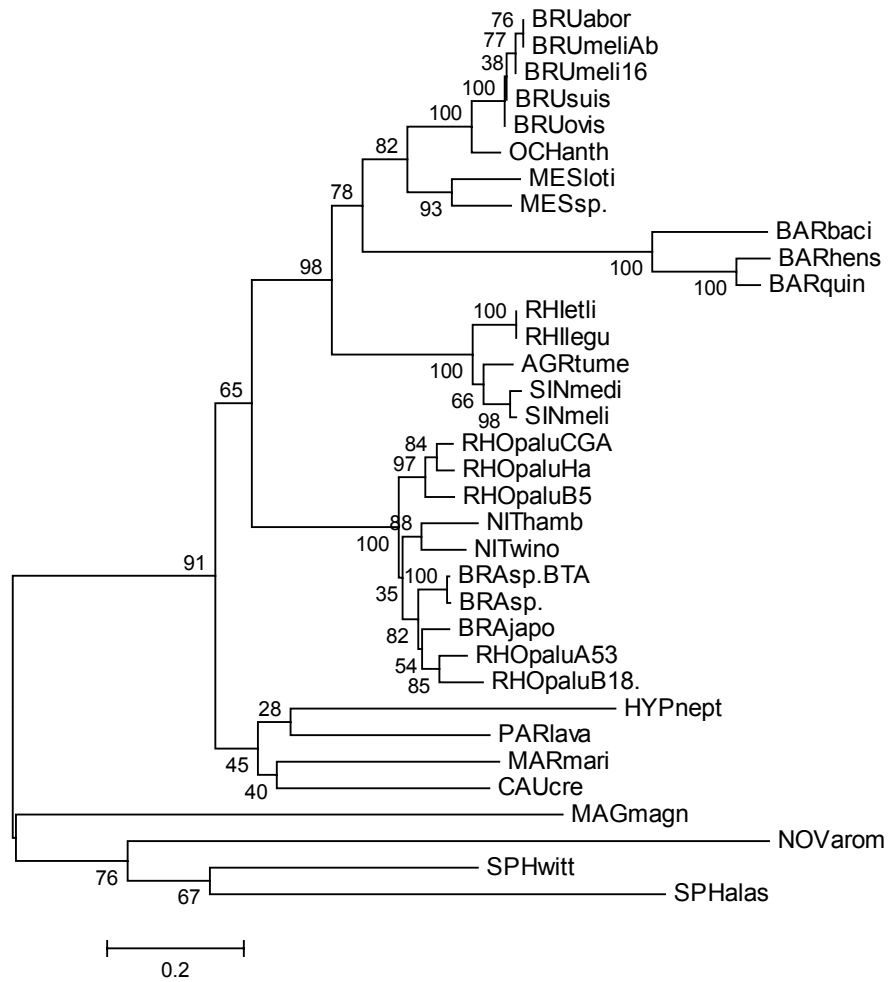

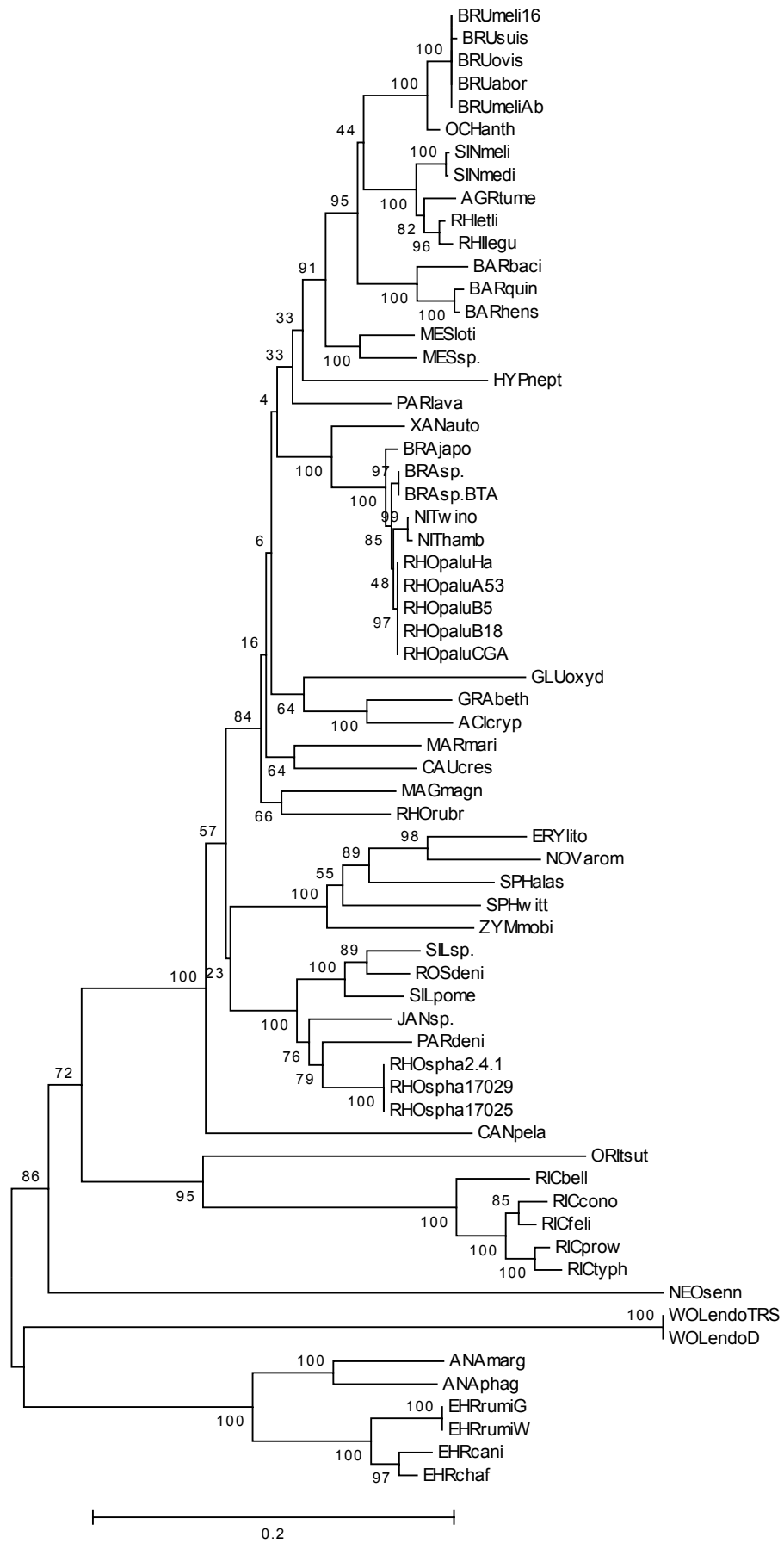

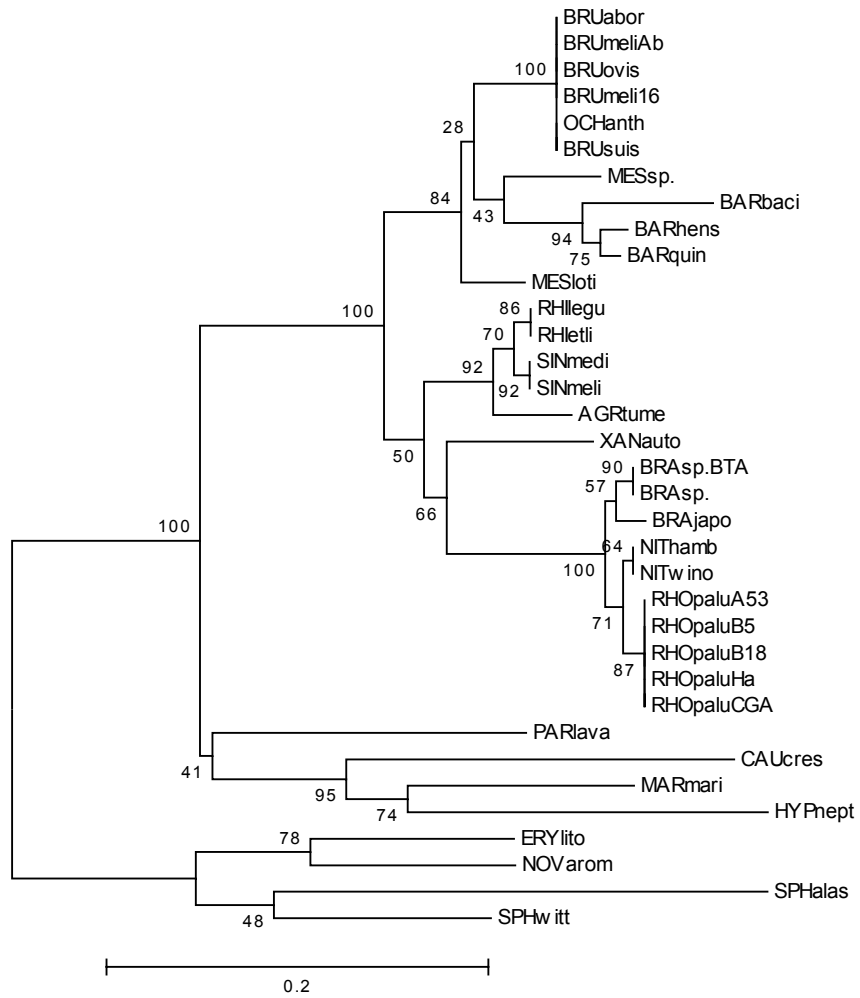

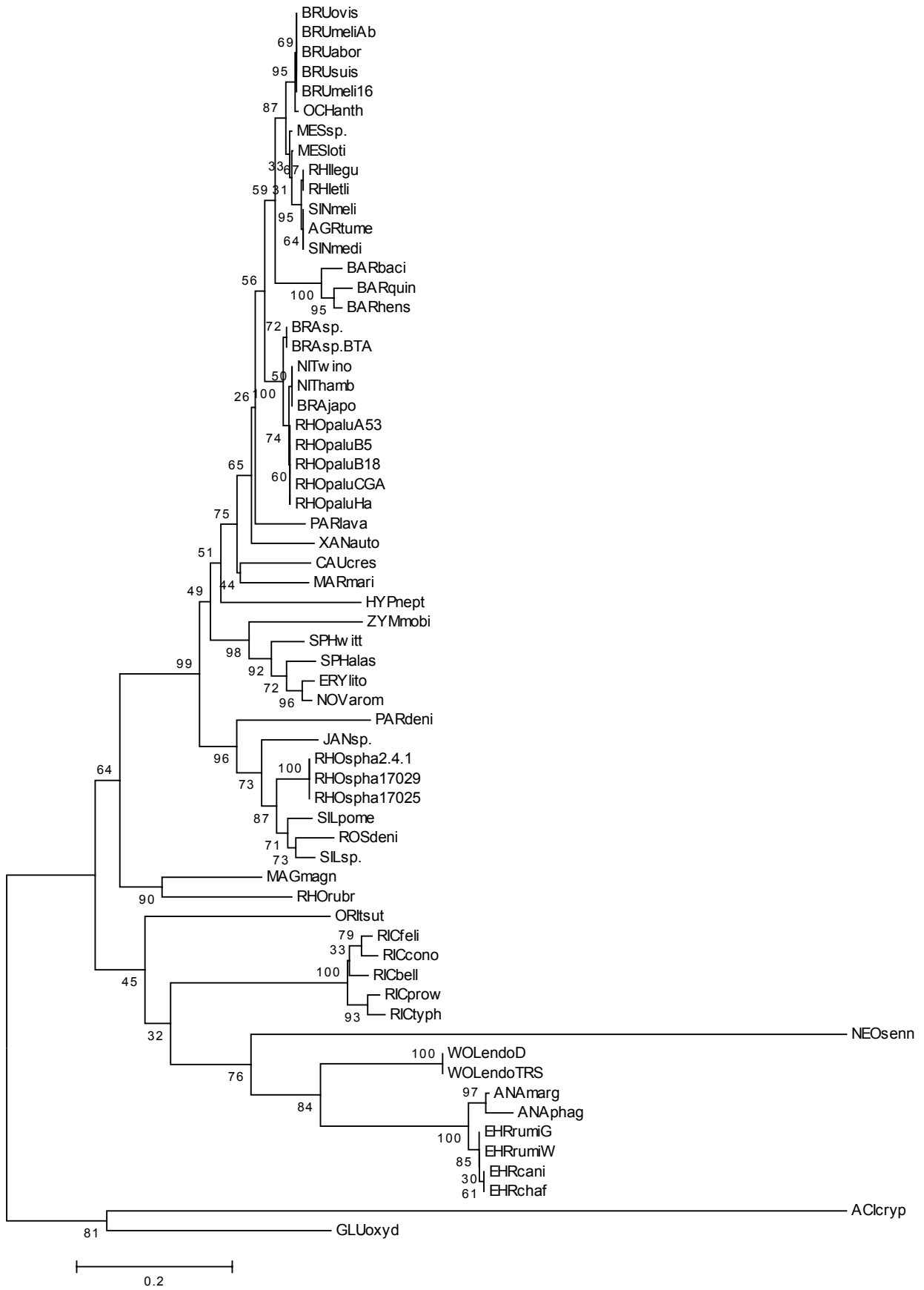

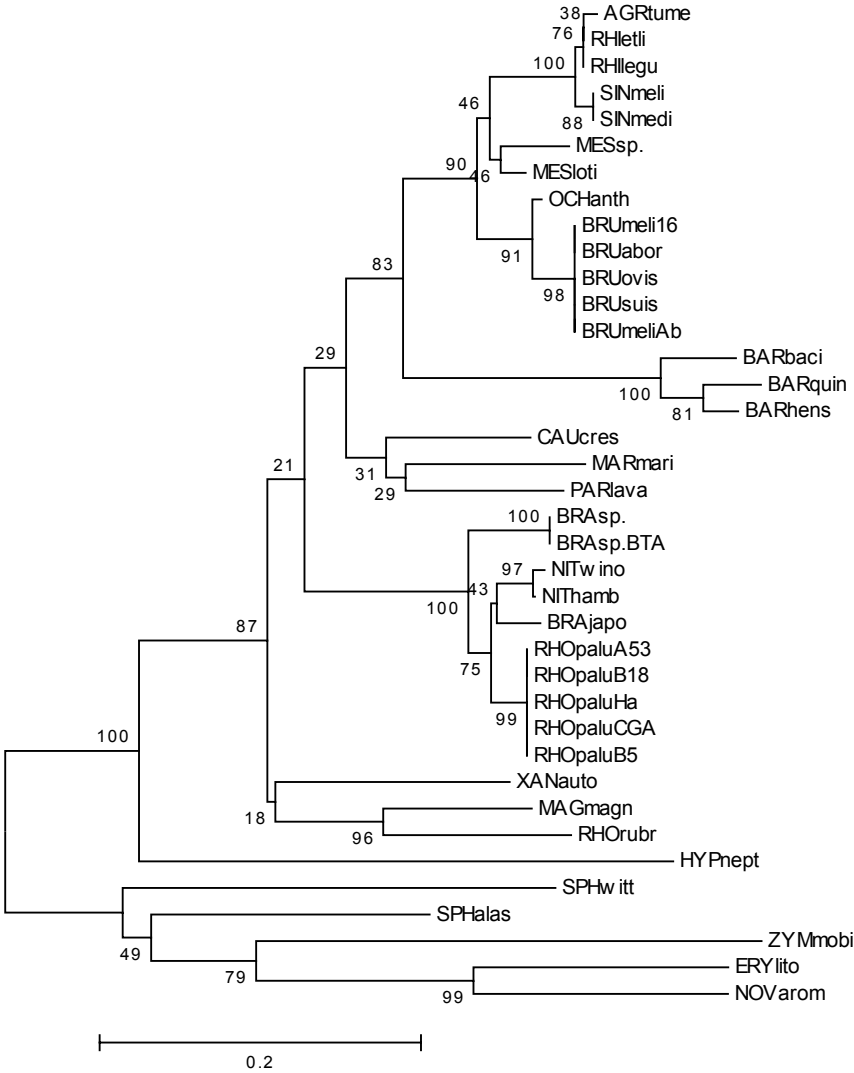

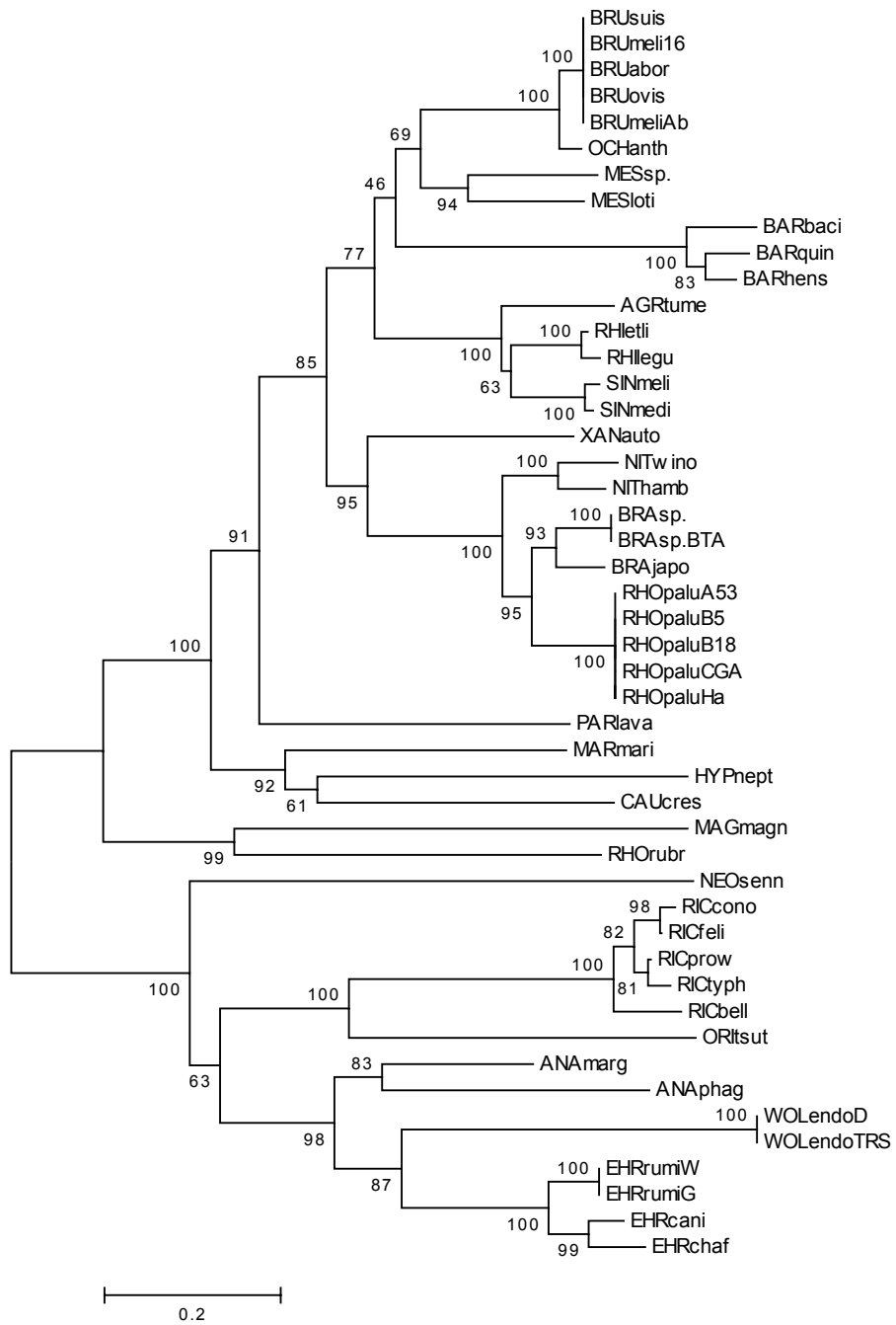

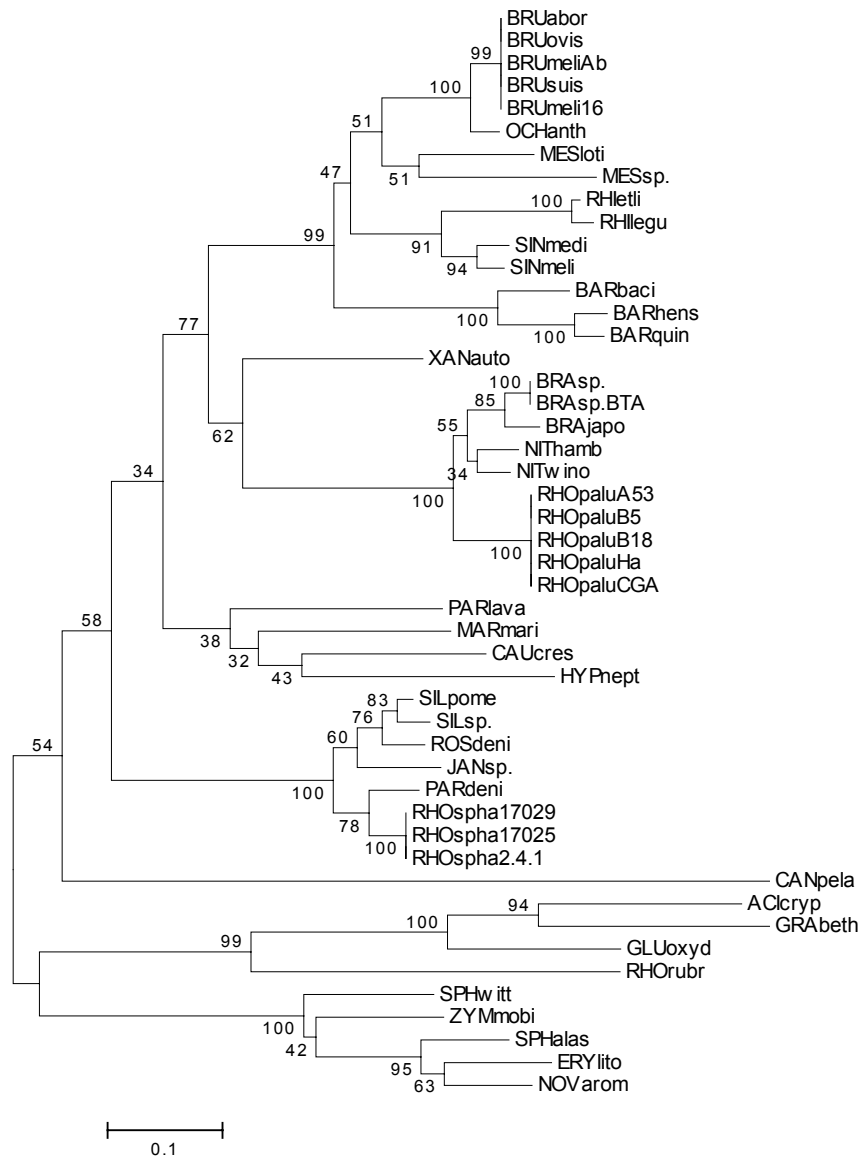

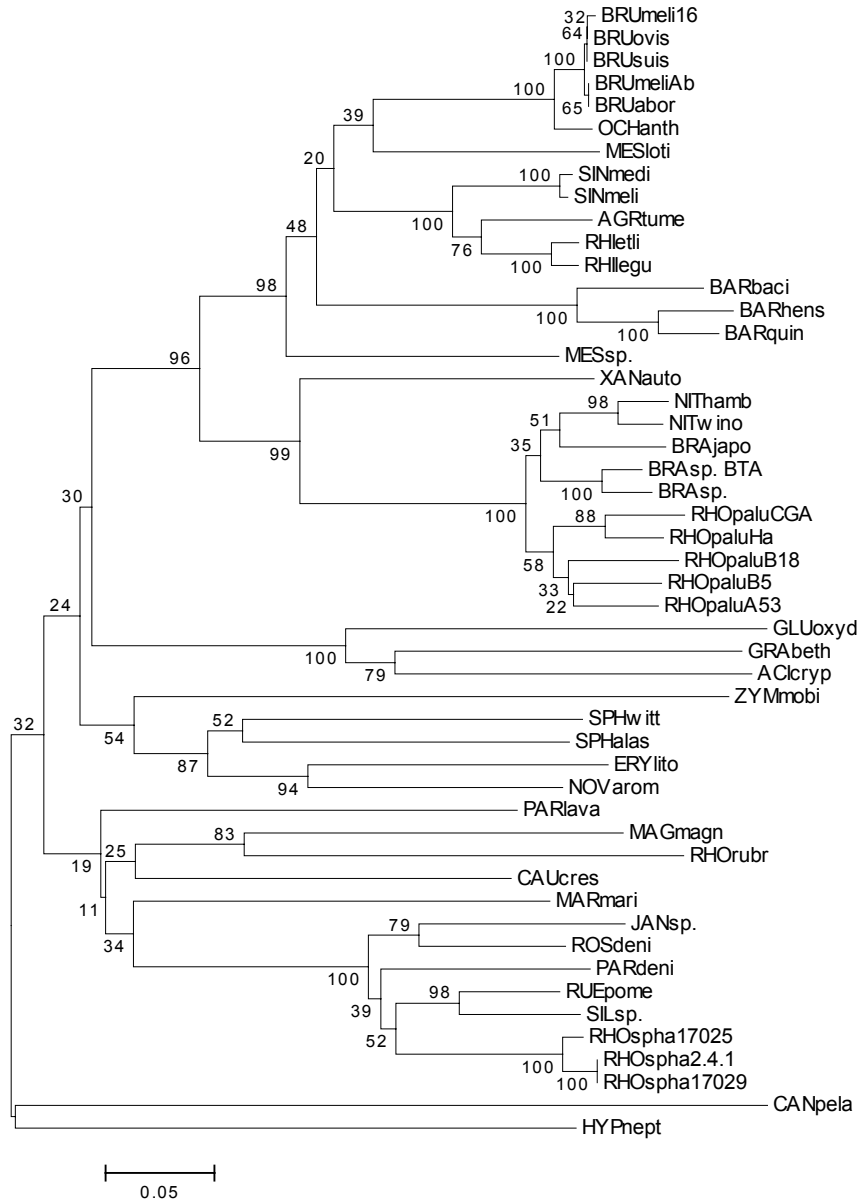

Supplement: Additional file 5 — Figure S2. Phylogenetic trees of cell cycle related proteins. [file 1752-0509-4-52-S5.PDF]

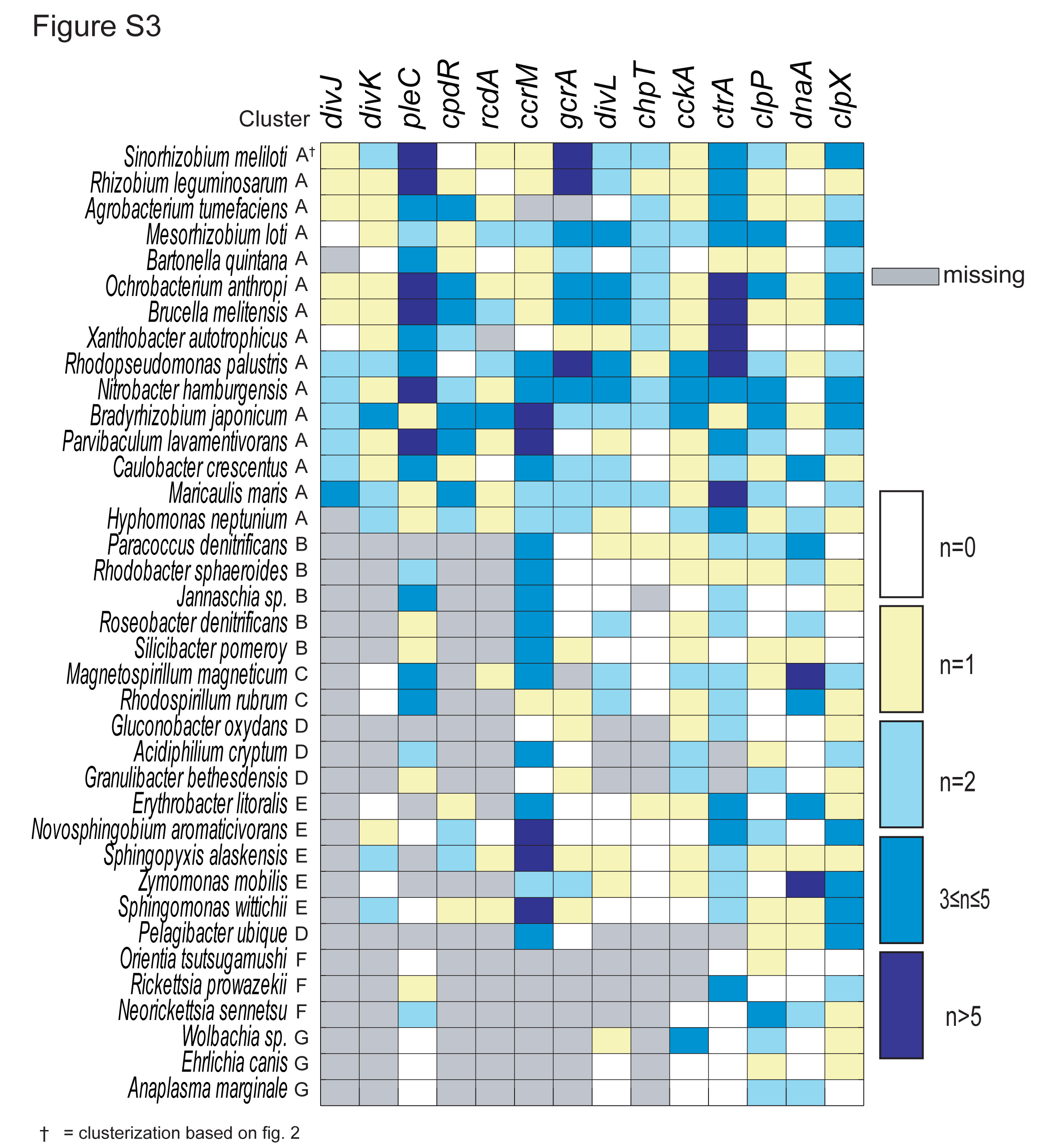

Supplement: Additional file 9 — Figure S3. Control of CcrM on cell cycle genes. [file 1752-0509-4-52-S9.JPEG]

Figure S4

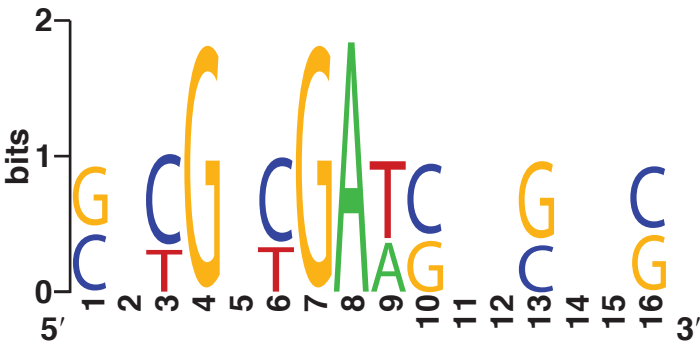

Supplement: Additional file 10 — Figure S4. Sequence logo of the putative GcrA motif. [file 1752-0509-4-52-S10.PDF]
